# Active-Sensing Epidermal Stretchable Bioelectronic Patch for Noninvasive, Conformal, and Wireless Tendon Monitoring

**DOI:** 10.34133/2021/9783432

**Published:** 2021-06-21

**Authors:** Sheng Shu, Jie An, Pengfei Chen, Di Liu, Ziming Wang, Chengyu Li, Shuangzhe Zhang, Yuan Liu, Jianzhe Luo, Lulu Zu, Wei Tang, Zhong Lin Wang

**Affiliations:** ^1^CAS Center for Excellence in Nanoscience, Beijing Key Laboratory of Micro-Nano Energy and Sensor, Beijing Institute of Nanoenergy and Nanosystems, Chinese Academy of Sciences, Beijing 100083, China; ^2^School of Nanoscience and Technology, University of Chinese Academy of Sciences, Beijing 100049, China; ^3^Center on Nanoenergy Research, School of Physical Science & Technology, Guangxi University, Nanning 530004, China; ^4^Institute of Applied Nanotechnology, Jiaxing, Zhejiang 314031, China; ^5^CUSPEA Institute of Technology, Wenzhou, Zhejiang 325024, China; ^6^School of Materials Science and Engineering, Georgia Institute of Technology, Atlanta, GA 30332-0245, USA

## Abstract

Sensors capable of monitoring dynamic mechanics of tendons throughout a body in real time could bring systematic information about a human body's physical condition, which is beneficial for avoiding muscle injury, checking hereditary muscle atrophy, and so on. However, the development of such sensors has been hindered by the requirement of superior portability, high resolution, and superb conformability. Here, we present a wearable and stretchable bioelectronic patch for detecting tendon activities. It is made up of a piezoelectric material, systematically optimized from architectures and mechanics, and exhibits a high resolution of 5.8 × 10^−5^ N with a linearity parameter of *R*^2^ = 0.999. Additionally, a tendon real-time monitoring and healthcare system is established by integrating the patch with a micro controller unit (MCU), which is able to process collected data and deliver feedback for exercise evaluation. Specifically, through the patch on the ankle, we measured the maximum force on the Achilles tendon during jumping which is about 16312 N, which is much higher than that during normal walking (3208 N) and running (5909 N). This work not only provides a strategy for facile monitoring of the variation of the tendon throughout the body but also throws light on the profound comprehension of human activities.

## 1. Introduction

Musculoskeletal diseases are one of the main causes of disability worldwide. In America, around 14 million people per year suffer from tendon, ligament, and joint injuries [1, 2]. Current clinical practice for tendon monitoring, such as magnetic resonance imaging (MRI) or ultrasound, could provide a snapshot of tendon density and inflammation but requires laboratory and high-cost equipment, reducing the possibility of real-time personal care [3, 4]. Implantable sensors could be used to assess real-time monitoring, allowing personalization of a rehabilitation protocol [5–7]. But the inconvenience of surgery, the strict requirements of biocompatible materials, and the need for sensors with high sensitivity and fast response time hindered its further development [3].

Noninvasive bioelectronic devices could be a promising approach to solve the above problems. The inertial system provides a low-cost, small-volume strategy for the tendon's epidermal testing, but the inevitable drift distortion affects its continuous monitoring [8]. The integrated sensor suit overcomes this properly, however, owing to the multijoint linkages; it can only give a synergetic motion result rather than that from a specific moving part [9–14]. Nowadays, the vibration property of the tendon has been detected and used as an evaluation parameter, which improves the accuracy of epidermal monitoring, but rigid vibration generators and accelerometers greatly reduce the wearability and comfort [15–17]. Epidemical wearable sensors offer a more promising approach due to their light weight, low cost, flexibility, and stretchability [18, 19]. We can obtain personal physical information by monitoring the heartbeat [20, 21], pulse [22, 23], sweat [24, 25], and even respiration [21, 26, 27] with the help of these sensors. But extracting physiological information from tendon activities is still rarely reported.

Based on mechanically induced polarization, piezoelectric devices have the characteristics of high sensitivity [13, 28] and self-powered ability [29, 30]. Besides, their inherent mechanical linear response makes them ideal sources for continuous dynamic tendon monitoring [31]. It can be found in the literature that thin-film devices are widely used in flexible electronics/optoelectronics, biomedical devices, energy storage, and conversion systems [32, 33]. Through structural design, such as origami [34], kirigami [35], bending [36], and winding [37], a thin piezoelectric film can be transformed to various two-dimensional (2D) or three-dimensional (3D) structures [38], which can give the devices new features such as stretchability and also enable them to deliver an accurate output in the required application conditions.

Here, we report an epidermal stretchable bioelectronic patch with a multiform-structured piezoelectric film. The patch can deform and stretch with the muscle and thus monitor tendon activities in real time. By integrating it with a micro controller unit (MCU), a tendon healthcare system is developed, which is able to collect and analyze motion data, display out on a smart phone, and finally serve as a reminder for personal daily exercising. Moreover, the whole flexible and stretchable packaging makes the entire system extraordinarily compliant. The rational use of commercial sports tape protects the device from the trouble of sweat and looseness as well. By attaching the patch on various muscles, we can precisely obtain tendon stretching motions around our body. And the Achilles's tendon (AT) is then systematically investigated. Our research has created a new epidermal monitoring system that could provide injury precaution and targeted treatments for tendons and generate personalized advice for individual recovery and professional sports training in the future.

## 2. Results

### 2.1. Multiform Epidermal Musculotendinous Sensing Unit

**Figure 1:**
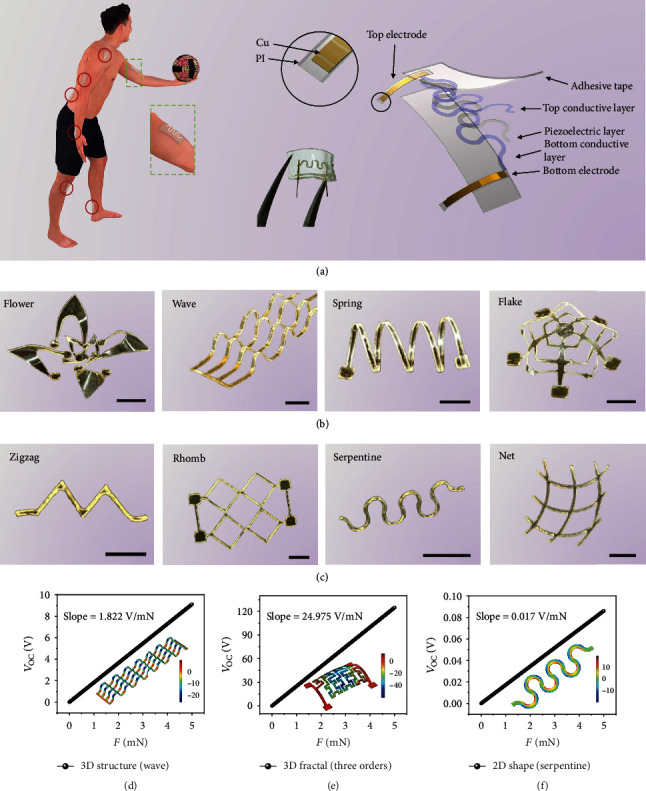
Multiform bioelectronic patch and 3D mesoscale piezoelectric frameworks. (a) Applications (left) and an exploded view of the patch (right). (b) Optical images of representative 3D mesoscale networks made up of PVDF, including origami (left), bending (middle left), winding (middle right), and kirigami (right). (c) Optical images of representative 2D structures by laser cutting, including zigzag (left), rhomb (middle left), serpentine (middle right), and net (right). Scale bars, 2 mm. (d–f) FEA results of typical structures: 3D structure (wave) (d), fractal structure (second order) (e), and 2D structure (serpentine) (f).

The musculotendinous sensing unit consists of functional piezoelectric films, metal conductors, and dielectric materials. Facile and mass fabrication processes such as laser cutting and printing are utilized.  Figure 1(a) illustrates the soft, stretchable musculotendinous bioelectronic patch applied all around the body. The exploded view shows the patch's stratified structure, consisting of a layer of piezoelectric polymer (polyvinylidene difluoride, PVDF, with a thickness of 28 *μ*m) and two layers of metal on the top (Cr/Ag, 10 nm/135 nm in thickness) and bottom (silver paste, 6 *μ*m in thickness) surfaces, as well as two layers of superelastic adhesive tapes (polyacrylate two-sided rubber, 1 mm), serving as the peripheral package. Electrical wires, made up of polyimide (PI, with a thickness of 110 *μ*m) and printed copper, are employed to connect with the sensing unit, ensuring the whole device's flexibility and electrical contact. An optical image of the fabricated patch with a total length of 8 mm is shown with a tweezer, and its open-circuit voltage under 20% stretching is presented in Supplementary Fig. 1.

Many researches proposed advanced folding technology for constructing 3D mesostructures for polymer devices [32, 36, 39, 40].  Figure 1(b) shows a variety of our sensing units with three-dimensional (3D) structures, including origami (left), bending (middle left), winding (middle right), and kirigami (right). These structures demonstrate the diversification of our fabrication approach. It begins with the use of a laser-cutting process to define a two-dimensional (2D) precursor made up of PVDF. Then, the as-fabricated precursor is transferred onto a prestrained elastomer covered with a thin layer of designed adhesive barrier (polyethylene terephthalate (PET) film with 50 *μ*m in thickness), so that the release of the prestrained substrate will transform the plane device into a well-defined resultant 3D architecture (Supplementary Fig. 2). Additional details are presented in Methods and in Supplementary Note 1. Such strategies establish approaches to fabricate 3D sensing units with various geometries, which are determined by the 2D precursor and the arrangement and magnitude of the prestrained elastomer substrate, as well as the profile of the adhesive barrier layer. To avoid the adhesion during large stretch, the fractal structure [40] is introduced and shown in Supplementary Fig. 3. With extremely fine curves and complex structures, it provides a relatively low adhesion between the PVDF films and the elastomer by virtue of the rough interface. After forming a 3D device, it can, therefore, smoothly switch between 2D and 3D geometries as stretching and releasing of the elastomer. Additionally, 2D-structured units could also exhibit excellent stretchability and meanwhile remain an ultralow thickness. As shown in Figure 1(c), various stretchable 2D sensing units are designed and manufactured, including zigzag (left), rhomb (middle left), serpentine (middle right), and net (right). Detailed fabrication information for 2D shapes is described in Methods and Supplementary Note 1.

Afterwards, all above structures are modeled (Supplementary Fig. 4-5), and finite element analysis (FEA) is conducted (Supplementary Note 2) to examine the devices' output performance. The forces applied here are tensile stresses acting on the cross sections at both ends of these structures. Figures 1(d)–1(f) illustrate FEA results of three representative structures. It reveals that these structures have excellent linearity, suitable for force sensing. More simulations about 2D and 3D structures can be found in Supplementary Fig. 6-7. The results indicate that the 3D structures have advantages in terms of tensile performance and voltage output (Supplementary Note 3), which is due to their relatively large precursors and sufficient prestrain. Nevertheless, 2D structures have the superiority in thickness and attachable comfort as wearable devices attached on tendons.

### 2.2. Design and Characteristics of the Epidermal Patch

**Figure 2:**
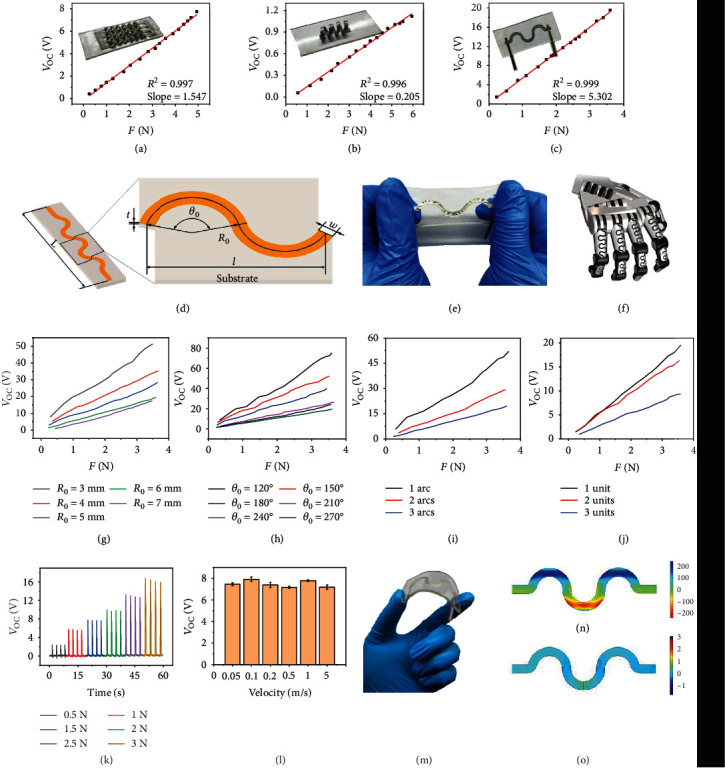
Design and optimization of the soft epidermal bioelectronic patch as well as its characteristics. (a–c) Experimental results of open-circuit voltage and linearity of different structures, including wave (a), spring (b), and serpentine (c). (d) Design diagram for the serpentine structure. (e) An optical imaging of the patch being stretched. (f) A potential application of the structure in a robotic hand. (g, h) Devices' output with various structure parameters: the radius (g) and the arc angle (h). (i, j) Devices' output with various unit connections, in series (i) and in parallel (j). (k) Diagram of open-circuit voltage under different forces. (l) The effect of different speeds on open-circuit voltage under a force of 1 N. (m) An optical image of the patch under bending. (n, o) FEA diagrams of surface potential when the patch is stretched and bent, respectively.

Here, three typical structures, with a total length of 50 mm, are designed and experimentally compared, including wave (Figure 2(a)), spring (Figure 2(b)), and serpentine (Figure 2(c)), and corresponding optical photographs are shown in the insets. They all have excellent linearity, but the open-circuit voltage of the 2D structure (serpentine) is significantly higher than that of the 3D (wave and spring) under the same force, which is contrary to previous simulations. This is because the above simulation model only contains a PVDF film, excluding the influence of the silicone substrate, while in practical experiments, the film will be restricted by the substrate. It is verified by a stretch FEA with a silicone substrate, shown in the Supplementary Fig. 8. Moreover, it also suggests that the silicone will introduce a lot of small deformations in the process of being stretched, so the stress distribution on the 2D units is more uniform. Therefore, 3D devices possess a thicker rubbery packaging, increasing the thickness of the whole sensor and reducing the attachability. Meanwhile, it shows a relatively lower output. Thus, the 2D serpentine structure is selected in our following experiments.

Figure 2(d) shows a fundamental shape of our 2D sensing unit with overall consideration of stretchability, flexibility, and compliance. It consists of two identical arc segments, defined by using arc angle (*θ*_0_), radius (*R*_0_), width (*w*), and thickness (*t*). The total length of the patch is set at 50 mm. An optical image of the patch being stretched is presented in Figure 2(e), and the designed maximum strain can reach more than 50%. Figure 2(f) shows a potential application of the structure in a robot hand. This fully flexible and stretchable sensor will also have great prospects in the robotic field.

Figures 2(g)–2(j) and Supplementary Fig. 9-16 present devices' output performance as a function of the increasing stretching force, with various devices' geometric parameters. Experiments are conducted by stretching the sensing units serially between a linear motor and an ergometer (Supplementary Fig. 17). Structures with different widths are firstly examined (Supplementary Fig. 9-10, with *R*_0_ = 6 mm, *θ*_0_ = 240°, *t* = 28 *μ*m, and unit number of 1.5). It can be seen that the output changes as the width varies. In this regard, the PVDF can be made narrow to ensure that the sensing component is cost-effective without lowering the output performance. The radius (*R*_0_) is another key device parameter. When it increases, the proportion of the arc part will increase accordingly, as shown in Supplementary Fig. 9, making the patch more stretchable. Figure 2(g) and Supplementary Fig. 11-12 (with *θ*_0_ = 240°, *w* = 3 mm, *t* = 28 *μ*m, and unit number of 1.5) plot the measuring results. It shows that as the radius increases, the open-circuit voltage decreases under the same tension, and the decreasing trend will gradually lessen. It could be attributed to that as the radius increases, the average deformation on per unit length in the longitudinal decreases, and thus, the output voltage decreases accordingly. And this is verified by the FEA results in Supplementary Fig. 13. Moreover, the angle of the arc (*θ*_0_) is found to increase the size of the PVDF and thus enhance the tensile properties. Results displayed in Figure 2(h) and Supplementary Fig. 14-15 (with *R*_0_ = 6 mm, *w* = 3 mm, *t* = 28 *μ*m, and unit number of 1.5) show that the open-circuit voltage decreases from 120° to 240° as the angle increases and rises slightly after 240°. It could be explained that as the angle increases, the deformation on per unit length in the longitudinal will decrease under the same tension, which is the main reason for the output's initial decrease. As the angle continues to increase, some spinodal appears. We speculate that the stretch-induced stress might be loaded on these spinodal, which increases the overall device's output to some degree. This could also be verified by Supplementary Fig. 16. Besides, we also checked the influence of the PVDF thickness (28, 52, and 110 microns) (Supplementary Fig. 18), and no obvious influence is obtained.

Subsequently, we studied the device performance with various connections. As shown in Figures 2(i) and 2(j) and Supplementary Fig. 19-22 (with *θ*_0_ = 240°, *R*_0_ = 6 mm, *w* = 3 mm, *t* = 28 *μ*m, and unit number of 1.5), arcs are connected in serials and parallel with a fixed overall length of 50 mm. It is found that the larger the arcs' number is, the lower the output is, under the same tension. The explanation is similar to that of angle-induced influence, which is as the number increases, the deformation on per unit length will decrease under the same tension, which is why the output decreases. But it is notable to mention that more arcs mean better tensile property, and reasonable tensile property is required to balance the output performance and the feasibility to apply on the human body. Figure 2(j) plots the results measured when the units are connected in parallel, which shows that as the number increases, the output decreases. It is attributing to that, under the same tension, more units in parallel will lead to lower deformation and thus lower output. Subsequently, the maximum strain of different radii and angles is measured (Supplementary Fig. 23), indicating that the maximum strain increases with the radius and angle. Therefore, sensors with a radius of 6 mm, arc angle of 180°, width of 3 mm, and 3 half units in serial, which could ensure a maximum strain over 30%, is selected as the standard device for the following experiments.

Figure 2(k) shows the open-circuit voltage of the device based on the above parameters, under diverse forces. It shows a good repeatability. Tiny mismatches might be due to the forces delivered by the linear motor being uneven. Afterwards, we evaluate the influence of different speeds with a tension of 1 N (Figure 2(l)). Waveforms are exhibited in Supplementary Fig. 24. The results show no significant difference, which is similar to FEA results under various frequencies (Supplementary Fig. 25). An excellent stretch-rebound ability is exhibited in Supplementary Fig. 26. After applying a constant strain of 20% for more than 20,000 cycles, the open-circuit voltage value increases by 10% (Supplementary Fig. 27). Moreover, when being applied to the human body, the patch will inevitably be bent (Figure 2(m)), suggesting that the output might be affected by bending. Supplementary Fig. 28 shows the output under a bending about 25% (more than 20°). It can be seen that, compared with the output under stretching, this output (about 0.3 V) is almost negligible. Figures 2(n) and 2(o) and Supplementary Movie S1 are the FEA diagram of surface potential distribution when the device is stretched and bent, respectively, verifying the above analysis.

### 2.3. Wearable Physical Property Sensing

**Figure 3:**
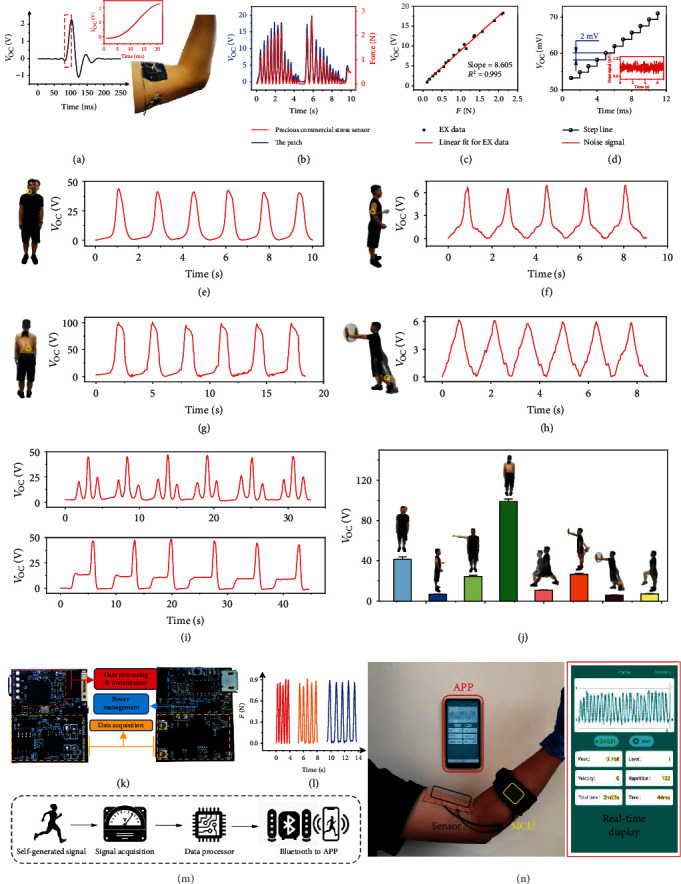
Wearable system validation of the patch on the tendon. (a) The response time of the patch, and the patch is serially connected with a commercial force sensor. (b) The synchronized diagram of the open-circuit voltage obtained by the patch and the corresponding stretching force measured by the commercial sensor. (c) Linear relationship between measured voltage and force in biceps. (d) A sensor-resolved voltage ladder diagram. (e–h) Open-circuit voltage of the patch sticking to the sternocleidomastoid (e), triceps brachii (f), erector spine (g), and gastrocnemius (h). (i) Swing and pause diagrams during the measurement of the sternocleidomastoid muscle. (j) Statistics diagram of signals obtained from various tendons all over the body. (k) An optical image of the MCU devised for portable purpose. (l) The signals of the patch at different frequencies on biceps brachii acquired from the MCU. (m) The operating procedures of the system. (n) Demonstration of applying the patch for recoding the arm's bending; the inset is a screenshot from the APP on the mobile phone.

The response time of the sensing unit, critical for real-time monitoring, is measured and shown in Figure 3(a) and its inset. The stretch-release cycle indicates a response time lower than 18 ms, which guarantees a timely feedback. A comparison between our patch and a commercial force sensor (resolution about 1 mN) is performed and shown in Figure 3(b) and Supplementary Fig. 29. The patch and the commercial sensor are connected in serial and attached to the upper arm. Excellent correspondence of the open-circuit voltage and the stretching force is observed. The linear fitting is shown in Figure 3(c), with *R*^2^ = 0.995 and slope = 8.605. In addition, we plot the loading curve with the time in Figure 3(d), which shows that the voltage increased gradually as the applied force increases, with an increase of about 2 mV. Furthermore, since the equipment noise is only 0.5 mV in our experiments, the theoretical measurement resolution of the patch can be calculated out, which is 5.8 × 10^−5^ N.

Figures 3(e)–3(h) and Supplementary Fig. 30 illustrate various motion signals obtained from our patch via attaching to the sternocleidomastoid, triceps brachii, erector spine, gastrocnemius, deltoid, anterior knee ligament, quadriceps femoris, and hamstring, respectively. Related actions are illustrated beside each diagram, and the patch location is marked via a yellow circle. It can be seen that, due to the thinness, flexibility, and stretchability, the patch can be placed all over the human body and monitor the tendon's activity in real time. The signal's peak value reflects the motion amplitude, which can serve as an indicator to prevent damage caused by excessive amplitude, and the signal's frequency and shape reflect the motion velocity and whether the action is distorted, etc. It can be seen from Figure 3(e), the frequency of the head swing is about 0.6 Hz, and the maximum amplitude is about 41.3 V (4.8 N). When there are external swings or stagnation actions (Figure 3(i)), the waveform varies accordingly (Supplementary Movie S2). We also plot a statistical diagram to give an overlook of the force amplitude of human daily motions, as shown in Figure 3(j). The erector spinae muscles show an apparent high value, meaning a large force during the according motion, which might explain why low back pain is the leading cause of global disability [1, 41].

Figure 3(k) exhibits a MCU with data acquisition, processing, and Bluetooth transmission modules. It is 3 cm × 4 cm in size, has a four-ply board craft, and utilizes the capacitive measuring principle to match the high impedance of the piezoelectric polymer. The incorporation of above-mentioned devices constitutes an active force measurement system which could actively sense and record deformation states of tendons and display it on the cellphone in real time. The signal of the patch obtained by our integrated circuit, during deformation with the biceps brachii, is plotted in Figure 3(l), showing a similar waveform with that measured by our experimental equipment of Keithley 6517, and it is displayed in Supplementary Movie S3. Figure 3(m) intuitively shows the operating procedures of the system. As vividly depicted in Figure 3(n), while a subject bends the arm, the deformation of the tendon is simultaneously recorded by the patch, transmitted by the MCU, and visualized in the mobile application (APP). The inserted image is an enlarged view of the APP, in which the top part exhibits real-time motions of the tendon, and six statistic parameters are listed below, including force peak, motion velocity, and repetitions. As shown in Supplementary Fig. 31, our sensing system can generate an intuitive feedback by grading and counting the force of each movement in the APP, providing useful advice for the profession sports actions or accurate medical rehabilitation.

### 2.4. Noninvasive Achilles Tendon (AT) Monitoring System

**Figure 4:**
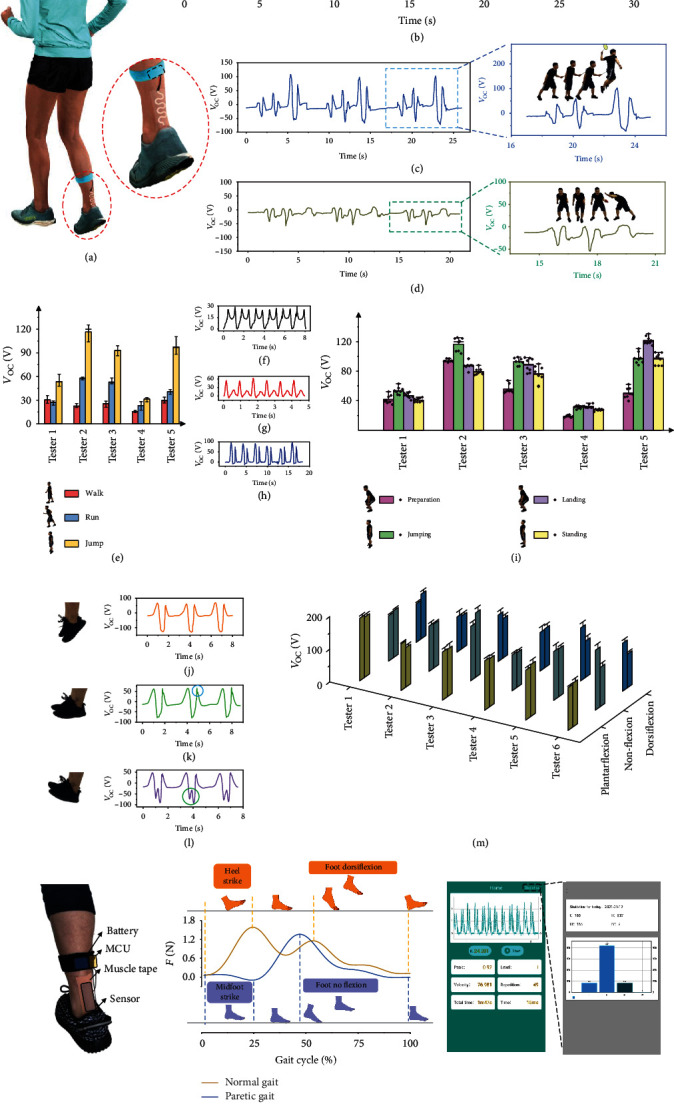
Noninvasive Achilles tendon monitoring. (a) Schematic diagram of wearing the system at the Achilles tendon. (b) The open-circuit voltage during basketball playing, including dribbling, running, and jump shooting. (c) The open-circuit voltage during badminton playing, including cross step and jump smash. (d) The open-circuit voltage of the lateral movement and swing in table tennis. (e) The statistic diagram of Achilles' tendon activity for people walking, running, and jumping. (f–h) The waveform of walking (f), running (g), and jumping (h). (i) The statistic diagram of open-circuit voltage under four different stages during jumping for different people, including preparation, jumping, landing, and standing. (j–l) Optical photographs of jumping with various postures and the waveforms: plantarflexion (j), nonflexion (k), and dorsiflexion (l). (m) A statistical analysis of the specific jumping actions: plantarflexion (yellow), nonflexion (green), and dorsiflexion (blue). (n) An optical image of the patch's fixation. (o) Using the bioelectronic system for gait analysis. (p) APP displays on a cellphone.

Ankle injury is one of the most common sports injuries, and it accounts for 40% of athletic injuries [42]. Achilles tendon (AT) management is required to reduce such injuries. Based on the previous analysis, we use this epidermal bioelectronic patch to monitor the AT activity in real time. The patch is attached directly on the epidermis between the fibula and the calcaneus and deforms as the tendon stretches and releases (Figure 4(a)). A MCU is connected and embedded in a fabric strap, for data collecting, processing, and transmitting to a smartphone via Bluetooth.

Figure 4(b) plots out the measured signals of a basketball player during dribbling, running, and jump shooting. It is interesting to find that the player shows a movement frequency of about 0.69 and 1.59 Hz when dribbling and running, respectively. And the voltage outputs are 35.4 and 65.2 V, respectively. Through the calculation in Supplementary Note 4, we can approximately get the forces applied on the AT, which are 3208 N and 5909 N. Notably, in a medical experiment that used physical methods to measure the force on the AT, the peak forces during walking and running are 3.9 and 7.7 times the body weight, respectively [43] (about 2869 N and 5660 N in our test), showing a good match. Moreover, during jump shooting, the tendon's loading time is about 0.495 s, with a recovering time of 0.31 s when landing. And it delivers the highest output of about 180 V, indicating that the greatest force on the AT is about 16312 N. This result might give a reasonable explanation for the fact that during basketball games, ankle injuries mostly occur at the moment of landing (45% of all AT injuries) [44]. In Figure 4(c), we analyze a set of typical badminton actions, including cross step and jump smash. An enlarged inset with decomposed actions is also presented. We can see that, during the cross step, the player alters his supporting leg, resulting in two peaks, about 7089 and 10868 N. Subsequently, if there comes a deep high service, a deep shot will result in a small voltage peak, while a clear ball will lead to a much larger peak. In addition, when smashing, a series of jumping peaks will appear (Supplementary Movie S4). Accordingly, jump smash will deliver the highest load on the Achilles tendon, which is about 15587 N in our test. As a comparison, in Figure 4(d), we illustrate the voltage signals for a table tennis player's AT. It can be found that, during swinging, and even killing, the force loaded on the AT is about 2519 N, which is similar to the forces of fast walking (about 2719 N). It might be why the main injury reason for table tennis fans is not focused on the Achilles tendon [45]. We perform the real-time monitoring on the continuous sports playing and show them in Supplementary Movie S5.

Furthermore, we utilize the patch to make a statistic on the loading forces of AT during personal normal exercises, including walking, running, and jumping. Subjects with various weights are selected, and the results are exhibited in Figure 4(e) and Supplementary Movie S6. The strain on the AT increases in the order of walking, running, and jumping. As we can see in Figures 4(f)–4(h), the forces are approximately 2704 N, 5438 N, and 10875 N, respectively. Subsequently, we study the jumping action in details. Figure 4(i) illustrates the statistic output values of different people during jumping, which is further divided into four stages: preparation, jumping, landing, and standing. As we can see, both preparation and standing actions deliver lower loads, while both jumping and landing actions deliver lager force. It is worth noting that, tester 4 shows much lower force than the others do, which is actually due to the wounds on his foot. In addition, we make a further analysis of the specific jumping actions, including plantarflexion, nonflexion, and dorsiflexion. Optical photographs and testing results are presented in Figures 4(j)–4(l). Clinically, an abnormal vertical ground reaction force will cause an explosive supination or inversion moment at the subtalar joint in a short time [46], which could be confirmed by the waveform. The peak value of the plantarflex jump is relatively round and smooth (Figure 4(j)), while the nonflex jump has a sharp peak (about 1982 N, circled in blue) when landing (Figure 4(k)) due to a shorter landing buffer distance (0.13 s), and the dorsiflex jump generates an extra peak (about 3688 N, Figure 4(l), circled in green). Further statistical analysis of different people jumping in Figure 4(m) can also prove the above phenomenon, in which the two bars with the same color represent stages of jumping and landing. As we can see, during plantarflex jumping, the force generated by jumping will be close to that by landing. If jumping abnormally (nonflexion or dorsiflexion), the difference can be found obviously. Database of forces generated by AT activities could provide important therapeutic support for AT recovery and posture correction. These results corroborate the potential of the epidermal patch as a high-performance monitor to realize real-time monitoring of AT.

Figure 4(n) shows an optical image of the fixation of our epidermal sensing patch, where the muscle tape and elastic strip serve as the auxiliary (Supplementary Note 5). As shown in Figure 4(o), Supplementary Fig. 31, and Movie S7, we utilize the real-time sensing system to compare a normal person's gait with that of the paretic patient (mimicking by bandage wrapping, which restricts the movement of the foot and thus leads to stiffness in the ankle, as shown in Supplementary Fig. 32) in one walking cycle. The yellow line represents the output signal of normal gait, and the blue stands for the paretic one. It is found that due to the restriction on the ankle, the paretic gait lacks fine movements, and thus, the signal exhibits only one peak, whereas that of the normal gait generates two peaks, corresponding to the contacting point between the foot and the floor, shifting forward. Details such as the peak force and the stepping velocity can be seen vividly in APP (Figure 4(p)). The wireless monitoring technology and accurate analysis capability enable the patch to be applied in the medical field promisingly.

## 3. Conclusion

We present a stretchable bioelectronic patch that can noninvasively monitor our bodies' tendons in real time. Multiform structures of piezoelectric polymers are designed, manufactured, and compared. On the basis of structural optimization, a serpentine patch with a precision of 5.8 × 10^−5^ N and *R*^2^ = 0.999 is selected. Additionally, the patch is applied on various tendons all throughout the body, and it shows excellent biological compatibility and universal applicability. Especially in measurements with the Achilles tendon, our patch delivers reliable analysis results, corresponds with clinical researches, and can detect action distortion in sports. In addition, we developed a tendon monitoring system, which is capable of real-time monitoring of the tendon's motions, proceeding collected data, and further offering feedback of motion evaluation. With this sensing system, we accomplish precise gait analysis and acquire classified statistics of daily exercise. We anticipate that this bioelectronic patch shows profound implications for the treatment and precautions of tendon diseases and opens a new door for targeted treatments that are widely advocated. In the future, the combination with machine learning and big data analysis can deliver visual evidence for in-depth analysis and understanding of the movement in our daily life.

## 4. Methods

### 4.1. Fabrication of 2D Piezoelectric Mesostructures

The fabrication of the stretchy 2D PVDF mesostructures began by preparing a PVDF film (28 *μ*m in thickness, MEAS) with silk-screen printing (6 *μ*m in thickness) on one side. Then, two sides of polyimide tape (PI, 25 *μ*m in thickness, Goldfinger) coated with silicone gel (75 *μ*m in thickness, Hon Shih Chiao) were tiled onto a Plexiglas substrate (2 mm in thickness, Creromem) of suitable size. Bubbles were removed. After that, the side of the PVDF film with silver paste was spread on the polyimide tape for graphic processing, and the laser-cutting machine (PLS6. 75) was adjusted to the appropriate focal length (0.5 mm), power (20%), and cutting speed (70%). The prepared mask, which is slightly smaller than the determined pattern, is placed on the PVDF film, and Cr/Ag (10 nm/135 nm in thickness) is deposited on the side covered with the mask by magnetron sputtering (Denton, Discovery 635). The mask plate is formed by laser cutting with a single fluorinated polyethylene terephthalate (PET) film (50 *μ*m in thickness). Under the environment of alcohol infiltration, the graphical PVDF was stripped by mechanical dissection, which completed the preparation of the 2D precursor. Adhesive VHB tape (polyacrylate two-sided rubber, 1 mm) is used to encapsulate and provide resilience. 2D bioelectronic patches can be obtained by stacking the VHB tape, bottom electrode, 2D precursor, top electrode, and VHB tape in sequence. These are done on release paper with an oily surface.

### 4.2. Fabrication of 3D Piezoelectric Mesostructures

Fabrication and transfer of 2D precursors followed the procedures for the 2D piezoelectric mesostructures described above. After obtaining the 2D precursor, prepare the rebound medium VHB tape, cover the tape with an adhesive barrier layer (PET, 50 *μ*m in thickness), cut the adhesive barrier layer on the tape into the desired shape through laser molding technology, and remove the excess part. This process requires a new definition of the focal length, cutting power, and cutting rate of the laser-cutting machine. A custom mechanical device is used to achieve an equally spaced stretching of the tape. The 2D precursors are then transferred to the stretched tape and connected by contact with a sticky surface. Finally, release the prestrain and transform the 2D precursors into 3D mesostructures.

### 4.3. Fabrication of the Flexible Electrode

The electrodes of the flexible bioelectric patch are prepared by using inkjet printing technology. Sputtering a copper film (50 nm) on a polyimide (PI) substrate (100 *μ*m in thickness), using an inkjet printer (Resist-Marking Printer Model SRP-3021) to print the desired electrode structure on the copper film, dissolving the copper without ink cover in ferric chloride solution, and then using another ink to wash off the printed ink, the resulting PI film is coated with graphical copper as the flexible electrode.

### 4.4. Electrical Output Measurement

The voltage signal of the multiform epidermal musculotendinous bioelectronic patch was acquired by a voltage preamplifier (Keithley 6517 System Electrometer). The signal is generated by the patch in series between the linear motor (Linmot, E1100) and the commercial stress sensor (Adbal, SH-5/10); signals from commercial sensors are directly read out after being processed internally. The whole measurement process of the system is fixed on the optical platform. The high-precision commercial sensor (Oruda, AT8301) used in series with the patch at biceps brachii determines the force by its own analog signal output using the built-in program. The MCU is the core of the whole wireless technology. It collects the charge quantity through the front circuit and converts it into voltage. After the digital-to-analog converter, the analog voltage amount is converted into a digital signal, which is then analyzed and processed by the low power Bluetooth module (TEXAS, CC2640R2F). These processes include calibration of voltage, zero return of initial value, and analysis of the amount of motion. Finally, the Bluetooth module transmits the data to the APP for real-time display. Considering the influence of age, height, body fat rate, etc., the correction coefficient *k* is introduced in the APP, which is a constant determined by the experiment to reduce the error.

## Data Availability

All data needed to evaluate the conclusions in the paper are present in the paper and/or the Supplementary Information. Additional data related to this paper may be requested from the authors upon reasonable request.

## Supplementary Materials

Supplementary 1 Supplementary note 1: detailed fabrication procedures for a representative 3D piezoelectric microsystem. Supplementary note 2: introduction to finite element analysis (FEA). Supplementary note 3: fundamental studies of the open-circuit voltage. Supplementary note 4: calculation of Achilles tendon (AT) force. Supplementary note 5: the auxiliary in in vitro physical property sensing. Table S1: comparison with existing wearable strain sensors. Figure S1: illustration of serpentine structure and its voltage output. (a) Optical imaging of our sensing unit on index figure. (b) Open-circuit voltage under 20% stretching of the unit in S1a. Figure S2: fabrication scheme for 3D piezoelectric mesostructure. Figure S3: 3D fractal designs. (a–c) Optical imaging of various fractal manufactures, including first- (a) and second-order (b) fractal curves and a (c) Hilbert geometry. Scale bar, 2 mm. Figure S4: 3D precursor of the structures shown in Figure 1. (a) Flower; (b) wave; (c) spring; (d) flake; (e–g) 3D precursor of the fractal structures, first order (e), second orders (f), and Hilbert geometry (g). Figure S5: FEA predictions of 3D structures. (a–c) FEA prediction of the buckled shape for the structures in Figure 1(b), including flower (a), wave (b), spring (c), and flake (d). (g–i) FEA prediction of the buckled shape for the fractal structures, first order (e), second orders (f), and Hilbert geometry (g). Figure S6: FEA results. (a–c) FEA of the 3D structures in Figure 1(b), including flower (a), spring (b), and flake (c). (d, e) FEA of the fractal structures with first order (d) and Hilbert geometry (e). (f) Comparison of three types of fractal structure. Figure S7: FEA of the 2D structures in Figure 1(c). (a) Zigzag; (b) rhomb; (c) net. Figure S8: FEA of the serpentine structure with silicone rubber substrate. (a) Strain distribution; (b) surface charge distribution; (c) FEA results; (d) experiment results. Figure S9: illustration of different width structural design. Figure S10: linear output graph with different width structure. Figure S11: illustration of different radius structural design in Figure 2(g). Figure S12: linear output graph with different radius in Figure 2(g). Figure S13: FEA results of different angles in Figure 2(g). (a–e) Surface potential distribution of various radii, including 3 mm (a), 4 mm (b), 5 mm (c), 6 mm (d), and 7 mm (e). (f) Comparison of surface potential changes with load at different radii. Figure S14: illustration of different angle structural design in Figure 2(h). Figure S15: linear output graph with different angles in Figure 2(h). Figure S16: FEA results of different angles in Figure 2(h). (a–e) Surface potential distribution of various angles, including 150° (a), 180° (b), 210° (c), 240° (d), and 270° (e). (f) Comparison of surface potential changes with load at different angles. Figure S17: quantitative mechanized measurement method of the patch. Figure S18: variation of open-circuit voltage with load under different thickness. (a–c) Linear output graph with different thickness structure, including 28 *μ*m (a), 52 *μ*m (b), and 110 *μ*m (c). (d) Comparison of open-circuit voltage changes with load at different thickness. Figure S19: illustration of different numbers of the arc structural design in Figure 2(i). Figure S20: linear output graph with different numbers of the arc structure in Figure 2(i). Figure S21: illustration of different numbers of the units' structural design in Figure 2(j). Figure S22: linear output graph with different numbers of the unit in Figure 2(j). Figure S23: relationship of radius and angle vs. strain to illustrate the stretchability of the structures in Figure 2. Figure S24: comparison of open-circuit voltages at various speeds. Figure S25: simulation diagram of frequency response characteristics under 1 N force. (a) Schematic diagram of force action. (b) Low frequency output under 1 N force. (c) Voltage vs. frequency graph. Figure S26: comparison chart of stretch and resilience performance. (a) Schematic of stretching. (b) Comparison of potential difference between stretch and rebound under various forces. Figure S27: cyclic measurement of the device under 20% of the tensile. Figure S28: comparison of open-circuit voltage between stretch and bending. (a) Schematic of bending. (b) Output comparison under 20% tension and compression; the red enlarged image shows the open-circuit voltage of the patch at 20% compression. Figure S29: diagram of the patch and commercial sensors in series. (a) Illustration of the connection between the patch and commercial sensors. (b) The patch and commercial sensors are fixed on the biceps for tandem measurement. Figure S30: open-circuit voltage of the patch sticking to the deltoid (a), quadriceps femoris (b), anterior knee ligament (c), and hamstrings (d). Figure S31: APP interface that can provide feedback information. Figure S32: simulation of paretic gait. (a) Diagram of gait simulation method. (b, c) APP screenshot of paretic gait monitoring.

Supplementary 2 Movie S1: FEA of surface stress and potential distribution.

Supplementary 3 Movie S2: sternocleidomastoid muscle test by second swing head.

Supplementary 4 Movie S3: bending the arm under different frequency.

Supplementary 5 Movie S4: different striking back signals for deep high service.

Supplementary 6 Movie S5: continuous motion measurement of various sports.

Supplementary 7 Movie S6: Achilles' tendon tests of daily activities.

Supplementary 8 Movie S7: gait analysis.
