# Supplementary material for: Active-Sensing Epidermal Stretchable Bioelectronic Patch for Noninvasive, Conformal, and Wireless Tendon Monitoring
Source: Research (Wash D C). 2021 Jun 21;2021:9783432. doi: 10.34133/2021/9783432 (PMC8244543; doi:10.34133/2021/9783432)
Supplement: Supplementary 1 — Supplementary note 1: detailed fabrication procedures for a representative 3D piezoelectric microsystem. Supplementary note 2: introduction to finite element analysis (FEA). Supplementary note 3: fundamental studies of the open-circuit voltage. Supplementary note 4: calculation of Achilles tendon (AT) force. Supplementary note 5: the auxiliary in in vitro physical property sensing. Table S1: comparison with existing wearable strain sensors. Figure S1: illustration of serpentine structure and its voltage output. (a) Optical imaging of our sensing unit on index figure. (b) Open-circuit voltage under 20% stretching of the unit in S1a. Figure S2: fabrication scheme for 3D piezoelectric mesostructure. Figure S3: 3D fractal designs. (a–c) Optical imaging of various fractal manufactures, including first- (a) and second-order (b) fractal curves and a (c) Hilbert geometry. Scale bar, 2 mm. Figure S4: 3D precursor of the structures shown in Figure 1. (a) Flower; (b) wave; (c) spring; (d) flake; (e–g) 3D precursor of the fractal structures, first order (e), second orders (f), and Hilbert geometry (g). Figure S5: FEA predictions of 3D structures. (a–c) FEA prediction of the buckled shape for the structures in Figure 1(b), including flower (a), wave (b), spring (c), and flake (d). (g–i) FEA prediction of the buckled shape for the fractal structures, first order (e), second orders (f), and Hilbert geometry (g). Figure S6: FEA results. (a–c) FEA of the 3D structures in Figure 1(b), including flower (a), spring (b), and flake (c). (d, e) FEA of the fractal structures with first order (d) and Hilbert geometry (e). (f) Comparison of three types of fractal structure. Figure S7: FEA of the 2D structures in Figure 1(c). (a) Zigzag; (b) rhomb; (c) net. Figure S8: FEA of the serpentine structure with silicone rubber substrate. (a) Strain distribution; (b) surface charge distribution; (c) FEA results; (d) experiment results. Figure S9: illustration of different width structural design. Fig [file 9783432.f1.docx]

**Supplementary Note 1: Detailed fabrication procedures for a representative 3D piezoelectric microsystem.**

**Preparing substrates:** First of all, a hard flat base is needed as the support for the original film material. The Plexiglas are selected (2mm in thickness) and cut into two 10x10cm squares by laser cutting machine, and clean the surface with alcohol and ionized water. Then, two sides of polyimide (PI, 25 μm in thickness) coated with silicone gel (75 μm in thickness) are applied flat to the surface of the plexiglass, and the bubbles are driven out afterwards. After that, one side of PVDF film (28μm in thickness) is wiped clean with alcohol and deionized water, and the other side is screen printed silver electrode (6 μm in thickness). After drying, the PVDF film is transferred to the PI tape on the plexiglas with the silver paste side facing down. In addition, the polyethylene terephthalate film (PET, 50 μm in thickness) is transferred to another plexiglass substrate attached with PI tape, which will be used for the masking.

**Define 2D patterns.** First, the 2D tile map of the required shape is designed by AutoCAD and imported into the laser cutting machine program. The focal height is set at 0.5mm, the power is 18%, and the cutting speed is 70%. Remove the excess and scrub the remaining PVDF edges with alcohol. The same procedure applies to the definition of the mask structure. The shape defined by the mask is slightly smaller than the PVDF shape, ensuring that the edges of the PVDF are completely covered by the mask. The focal height is set at -0.5mm, the power is 22%, and the cutting speed is 70%. Remove excess cut material and mechanically peel the mask off the tape. The mask is transferred to the defined PVDF and aligned with it, and the mask is fixed by the double-sided adhesive PI tape below the PVDF. Keep the whole surface clean and electroplate it with magnetron sputtering apparatus (Cr/Ag, 10nm/135nm in thickness). The two electrodes are prepared separately because laser cutting is cut by melting, which causes the top and bottom electrodes to dissolve and contact, resulting in a short circuit. Finally, the 2D structure and the substrate were separated by mechanical stripping under alcohol infiltration.

**Form 3D folds.** Prepare a piece of VHB tape of appropriate size (double-sided tape, 60x30x1mm, 3M4905), and stick it gently on the plexiglas cleaned with alcohol to ensure that the surface is as smooth as possible after sticking. The clean PET film (50 μm in thickness) is then transferred to the VHB tape. Adjust the laser cutting machine to 1.5mm focal height, 15% power and 65% speed, and cut the same position twice. The cut pattern is also a special pattern previously designed with AutoCAD. For example, when preparing wave patterns, the narrow strip for adhesion should be removed and the wide strip for adhesion be retained. The VHB tape is mechanically removed under alcohol immersion and transferred to a specially designed mechanical tensile device. To ensure uniformity of drawing, the device is designed to be scalable with multiple diamond buckles. Under the action of fixed directional force, the VHB tape is stretched by 60% and the mechanical device is fixed. The 2D precursor defined previously is transferred to the stretched VHB surface and aligned with the shape of the adhesive layer. And it is fixed by the surface viscosity of the VHB tape. The prestress is then released, allowing it to bounce back to its original position, which completes the transition from 2D precursors to 3D structures. Finally, the barrier layer is removed by means of extraction.

**Supplementary Note 2:** **Introduction to** **Finite element analysis (FEA)**

FEA is conducted to simulate the force distribution and surface potential changes acting on the design structure in as much detail as possible, which is helpful for us to understand and optimize the structure. Here the commercial software COMSOL Multiphysics (version 5.4, standard) was chosen to execute FEA. For more accurate analysis of the force, the models used by FEA were all 3D models that have been constructed by SolidWorks (version 2016, standard). In the process of model simulation with a base, silicone rubber is introduced as a stretching medium, fixed with the PVDF through a connection, and stretched with it. The materials used in the simulation are provided by COMSOL's built-in material library. The properties of materials including Young's modulus and Poisson's ratio are calculated from the material library given the structure and elastic matrix.

Materia information: Relative dielectric constant: {7.4, 9.3, 7.6}; Density: 1780[kg/m^3]; Elastic matric: {3.8e+09[Pa], 1.9e+09[Pa], 3.8e+09[Pa], 0.9e+09[Pa], 0.9e+09[Pa], 1.2e+09[Pa], 0[Pa], 0[Pa], 0[Pa], 7e+08[Pa], 0[Pa], 0[Pa], 0[Pa], 0[Pa], 9e+08[Pa], 0[Pa], 0[Pa], 0[Pa], 0[Pa], 0[Pa], 9e+08[Pa]}; Coupled matric: {0[C/m^2], 0[C/m^2], 0.024[C/m^2], 0[C/m^2], 0[C/m^2], 0.001[C/m^2], 0[C/m^2], 0[C/m^2], -0.027[C/m^2], 0[C/m^2], 0[C/m^2], 0[C/m^2], 0[C/m^2], 0[C/m^2], 0[C/m^2], 0[C/m^2], 0[C/m^2], 0[C/m^2]}.All other necessary information such as Young’s module and Poisson’s ratio comes from Materia Library.

Firstly, a three-dimensional CAD model was imported into COMSOL based on piezoelectric analysis. The whole model material is set as Polyvinylidene fluoride (PVDF). In the option of solid mechanics, select "from material" for all mechanical properties. A "fixed constraint" is applied to the section at one end of the model, and a variable load (parameterized) is set at the other end. The "designated displacement" is set to 0 for the lower boundary to ensure that no changes occur in multiple directions during the stretching process. One end of the material is then "grounded" and the electrostatic properties are also " from material". The hydrodynamic grid is divided into free tetrahedron. Finally, parameterized setting and calculation are carried out.

**Supplementary Note 3: Fundamental studies of the open-circuit voltage**

In the basic analysis, the open circuit voltage obtained during the stretching process is derived from the relationship between the piezoelectric potential and the stress calculated by the FEA. Under small field conditions, as a sensor, non-sensing parts such as drivers can be ignored and simplified^1^. Its electric displacement vector $D_{i}$ can be expressed as:

$$\begin{aligned} \boldsymbol{D}_{\boldsymbol{i}}=\boldsymbol{e}_{iq}\boldsymbol{\varepsilon}_{q}+\boldsymbol{k}_{ik}\boldsymbol{E}_{k}\boldsymbol{\#}\left( s1 \right) \end{aligned}$$

where $e_{iq}$ is the elastic compliance, $\varepsilon_{q}$ is the stress vector, the $k_{ik}$ is the dielectric permittivity, and $E_{k}$ is the applied electric field vector. Here, 1, 2, 3 respectively represent the length, width and thickness direction. Assuming that the PVDF film used is polarized along the thickness direction (3-axis) and the 1-axis and 2-aixs are in the plane of the sheet. The $e_{iq}$ matrix can be expressed as:

$$\begin{aligned} \boldsymbol{e}_{iq}= \left[ \begin{matrix} 0 & 0 & 0 \\ 0 & 0 & 0 \\ e_{31} & e_{32} & e_{33} \end{matrix} \begin{matrix} 0 & e_{15} & 0 \\ e_{24} & 0 & 0 \\ 0 & 0 & 0 \end{matrix} \right]\#\left( s2 \right) \end{aligned}$$

And the permittivity matrix is

$$\begin{aligned} \boldsymbol{k}_{ik}=\left[ \begin{matrix} k_{11} & 0 & 0 \\ 0 & k_{22} & 0 \\ 0 & 0 & k_{33} \end{matrix} \right]\boldsymbol{\#}\left( S3 \right) \end{aligned}$$

The sensor is exposed to the stress field, generates a corresponding stress charge, and can be measured. In the case of a sensor, when the applied external electric field is zero, Equation (s1) gets into

$$\begin{aligned} \boldsymbol{D}_{\boldsymbol{i}}=\boldsymbol{e}_{iq}\boldsymbol{\varepsilon}_{q}\boldsymbol{\#}\left( S4 \right) \end{aligned}$$

Which can be rewritten as

$$\begin{aligned} \left[ \begin{matrix} D_{1} \\ D_{2} \\ D_{3} \end{matrix} \right]=\left[ \begin{matrix} 0 & 0 & 0 \\ 0 & 0 & 0 \\ e_{31} & e_{32} & e_{33} \end{matrix} \begin{matrix} 0 & e_{15} & 0 \\ e_{24} & 0 & 0 \\ 0 & 0 & 0 \end{matrix} \right]\left[ \begin{matrix} \begin{matrix} \sigma_{1} \\ \sigma_{2} \\ \sigma_{3} \end{matrix} \\ \sigma_{4} \\ \begin{matrix} \sigma_{5} \\ \sigma_{6} \end{matrix} \end{matrix} \right]\#\left( S5 \right) \end{aligned}$$

where $\sigma_{1}-\sigma_{6}$ is the stress obtained from the FEA. This formula clarifies that the electric displacement vector is the result of the piezoelectric effect produced by the stress field acting on the piezoelectric material^2, 3^. According to the relationship between the electric displacement vector and the amount of charge generated, we can get

$$\begin{aligned} q=\iint\left[ \begin{matrix} D_{1} & D_{2} & D_{3} \end{matrix} \right]\left[ \begin{matrix} dA_{1} \\ {dA}_{2} \\ dA_{3} \end{matrix} \right]\#\left( S6 \right) \end{aligned}$$

Where ${dA}_{1}{, dA}_{2}{, dA}_{3}$are the components of the electrode area in the 2-3, 1-3, and 1-2 planes respectively. A typical thin film can be treated as a parallel plate capacitor, whose capacitance$C_{p}$ can be defined as

$$\begin{aligned} C_{p}=\frac{e_{33}l_{a}w_{a}}{t_{a}}\#\left( S7 \right) \end{aligned}$$

Where$l_{a}, w_{a}$and $t_{a}$ respectively represent the length, width and thickness of the piezoelectric film. Therefore, the open-circuit voltage can be got

$$\begin{aligned} V_{oc}=\frac{q}{C_{p}}=\frac{t_{a}}{e_{33}l_{a}w_{a}}\iint\left[ e_{15}\sigma_{5}{dA}_{1}+e_{24}\sigma_{4}{dA}_{2}+\left( e_{31}\sigma_{1}+e_{32}\sigma_{2}+e_{33}\sigma_{3} \right){dA}_{3} \right]\#\left( S8 \right) \end{aligned}$$

Considering that when 3D structures are straightened, the direction of stretching is always determined along the 1 direction. Under this circumstance, combining Equations S6 we can get

$$\begin{aligned} q_{1}=d_{31}Y_{a}w_{a}\int_{0}^{l_{a}} \varepsilon_{1}dx\#\left( S9 \right) \end{aligned}$$

where$Y_{a}$ is the Young’s modulus of the PVDF film, and $d_{31}$ is piezoelectric coefficient of 1-direction. And the open-circuit voltage can also be acquired

$$\begin{aligned} V_{oc1}=\frac{q_{1}}{C_{p}}=\frac{d_{31}Y_{a}t_{a}}{e_{33}l_{a}}\int_{0}^{l_{a}} \varepsilon_{1}dx\#\left( S10 \right) \end{aligned}$$

**Supplementary Note 4:** **Calculation of** **Achilles tendon (AT) force**.

In the experiment of Fig.3c, we have clarified the power-to-electricity conversion relationship of the patch (Slope=8.605V/N), and the force received by the patch can be easily obtained by this equation:

$$\begin{aligned} F_{patch}=\frac{V_{measure}}{8.605}\#\left( S11 \right) \end{aligned}$$

where $F_{patch}$ is the force on the patch, $V_{measure}$ is voltages the patch generated. In this experiment, we obtained the Young's modulus of the patch by the following equation:

$$\begin{aligned} E_{patch}=\frac{\sigma}{\varepsilon}=\frac{F_{patch}L}{A_{patch} \Delta L}\#\left( S12 \right) \end{aligned}$$

Where E is the Young's modulus, $\sigma$ is the stress, $\varepsilon$ is the strain, $L$ is the total length of the patch, $A$ is the sectional area and $\Delta L$ is the variation when stretching.

When attached to the epidermis outside the AT, the patch is parallel to the AT, and the two will have the proportional displacement while deforming with muscle activities. Based on this, we can easily get the relationship between the forces on the two:

$$\begin{aligned} \varepsilon_{patch}=\frac{\sigma_{patch}}{E_{patch}}=\frac{\sigma_{AT}L_{patch}}{E_{At}L_{AT}}\#\left( S13 \right) \end{aligned}$$

Which can be rewritten as:

$$\begin{aligned} F_{AT}=\frac{A_{AT}E_{AT}L_{AT}}{A_{patch}E_{patch}L_{patch}}F_{patch}\#\left( S14 \right) \end{aligned}$$

Here, the Young's modulus and sectional area of Achilles tendon are the mean value of the statistical data, *A_AT_ =* 51.5 *mm^2^; E_AT_ =* 0.75 *GPa; L_AT_ =* 50 *mm; A_patch_ =* 30 *mm^2^; E_patch_ =*1.651 *MPa; L_patch_ =* 50 *mm* and the body weight of the selected tester is 75kg^4, 5^.

**Supplementary Note 5: The auxiliary in in vitro physical property sensing**.

The muscle tape is mainly used for auxiliary fixation, including muscle activities throughout the body and movement of the Achilles tendon. Muscle tapes have been used as an intermediary in series contrast to commercial sensors. Open at both ends of the muscle tape, connect one side of clear cellophane with adhesive (3M, Scotch), and connect to a ring before connecting to a commercial sensor. In addition, to adapt to the constantly changing environment during exercise, the MCU is embedded in a fabric ankle guard, which can be worn with the guard while avoiding the contamination of the circuit with sweat stains. The MCU and the sensor are wired and then communicate with the mobile APP using Bluetooth. In order to simulate the inconvenient state of foot injury, the bandage is used to fix the ankle, and the ring binding method is the figure eight winding method, which is to do the figure eight winding from the outside of the ankle bone to the top of the foot, to the bottom of the foot, to the outside of the arch of the foot, to the inside of the ankle bone to the Achilles tendon from the inside out.

**Table S1: Comparison with existing wearable strain sensors.**

| Type | Reference | Active Materia | Sensitivity | Durability (Cycle) | Response Time (ms) | Signal Output | Stretchability（%） |
| --- | --- | --- | --- | --- | --- | --- | --- |
| Capacity | ^6^ | Ecoflex | 0.57–1.62  Mpa^–1^ | 100 | 40 |  | 50 |
|  | ^7^ | Polyethylene | 0.17 kPa–1 | 100 |  |  | 55 |
|  | ^8^ | PDMS | GF = 0.97 | 10000 | 100 |  | 100 |
| Piezoresistive | ^9^ | rGO/TPU fibrous | GF = 598 | 500 | 160 |  | 150 |
|  | ^10^ | Ionic covalent hydrogel |  | 200 | 800 |  | 1700 |
|  | ^11^ | Carbonized sponge/  Ecoflex | GF = 4.25–18.42 | 1000 | 100 |  | 600 |
| piezoelectric | ^12^ | P(VDF-TrFE) |  | 4000 |  | 1.4 V | 30 |
|  | ^13^ | PbZr0.52Ti0.48O3 (PZT) | 0.197N/V | 10000 |  | 20 V | 200 |
|  | **Our patch** | PVDF | 8.605N/V | 20000 | 18 | 180 V+ | 100 |
| Others | ^14^ | Poly(vinyl alcohol) nanofibers |  | 2700 | 70 |  | 100 |
|  | ^15^ | Metal/Polymer | 0.01N/cm | 2000 |  |  | 57.1 |
|  | ^16^ | Aggregation-induced  emission compound | 34 mV Pa−1. | 5800 | 60-90 |  | 160 |

**Reference:**

1. Sirohi, J. & Chopra, I. Fundamental Understanding of Piezoelectric Strain Sensors. *Journal of Intelligent Material Systems and Structures* **11**, 246-257 (2016).

2. Wang, Z.L. Piezotronics and Piezo‐Phototronics. *Wiley Encyclopedia of Electrical and Electronics Engineering*, 1-18 (2001).

3. Wang, Z.L. (Georgia Institute of Technology, 2011).

4. Martin, K.D., Wake, J., Dawson, L. & Van Buren, J.P. Cross-sectional Area of the Achilles Tendon in a Cohort of Elite Military Warriors Using Standard Ultrasound Techniques. *Mil Med* **183**, e744-e747 (2018).

5. Maganaris, C.N., Narici, M.V. & Maffulli, N. Biomechanics of the Achilles tendon. *Disabil Rehabil* **30**, 1542-1547 (2008).

6. Yao, S. & Zhu, Y. Wearable multifunctional sensors using printed stretchable conductors made of silver nanowires. *Nanoscale* **6**, 2345-2352 (2014).

7. Lei, Z., Wang, Q., Sun, S., Zhu, W. & Wu, P. A Bioinspired Mineral Hydrogel as a Self-Healable, Mechanically Adaptable Ionic Skin for Highly Sensitive Pressure Sensing. *Adv Mater* **29** (2017).

8. Cai, L. et al. Super-stretchable, transparent carbon nanotube-based capacitive strain sensors for human motion detection. *Sci Rep* **3**, 3048 (2013).

9. Li, G. et al. Aligned flexible conductive fibrous networks for highly sensitive, ultrastretchable and wearable strain sensors. *Journal of Materials Chemistry C* **6**, 6575-6583 (2018).

10. Sun, H. et al. Highly Stretchable, Transparent, and Bio‐Friendly Strain Sensor Based on Self‐Recovery Ionic‐Covalent Hydrogels for Human Motion Monitoring. *Macromolecular Materials and Engineering* **304** (2019).

11. Yu, X.G. et al. A wearable strain sensor based on a carbonized nano-sponge/silicone composite for human motion detection. *Nanoscale* **9**, 6680-6685 (2017).

12. Lee, J.H. et al. Highly stretchable piezoelectric-pyroelectric hybrid nanogenerator. *Adv Mater* **26**, 765-769 (2014).

13. Chou, X. et al. All-in-one filler-elastomer-based high-performance stretchable piezoelectric nanogenerator for kinetic energy harvesting and self-powered motion monitoring. *Nano Energy* **53**, 550-558 (2018).

14. Wang, X. et al. A Highly Stretchable Transparent Self-Powered Triboelectric Tactile Sensor with Metallized Nanofibers for Wearable Electronics. *Adv Mater* **30**, e1706738 (2018).

15. Hussain, A.M. et al. Metal/Polymer Based Stretchable Antenna for Constant Frequency Far-Field Communication in Wearable Electronics. *Advanced Functional Materials* **25**, 6565-6575 (2015).

16. Bu, T. et al. Stretchable triboelectric–photonic smart skin for tactile and gesture sensing. **30**, 1800066 (2018).


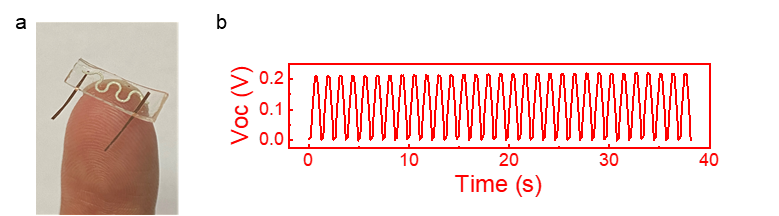


**Supplementary Figure 1. Illustration of serpentine structure and its voltage output. a**. Optical imagine of our sensing unit on index figure. **b**. Open-circuit voltage under 20% stretching of the unit in S1a.


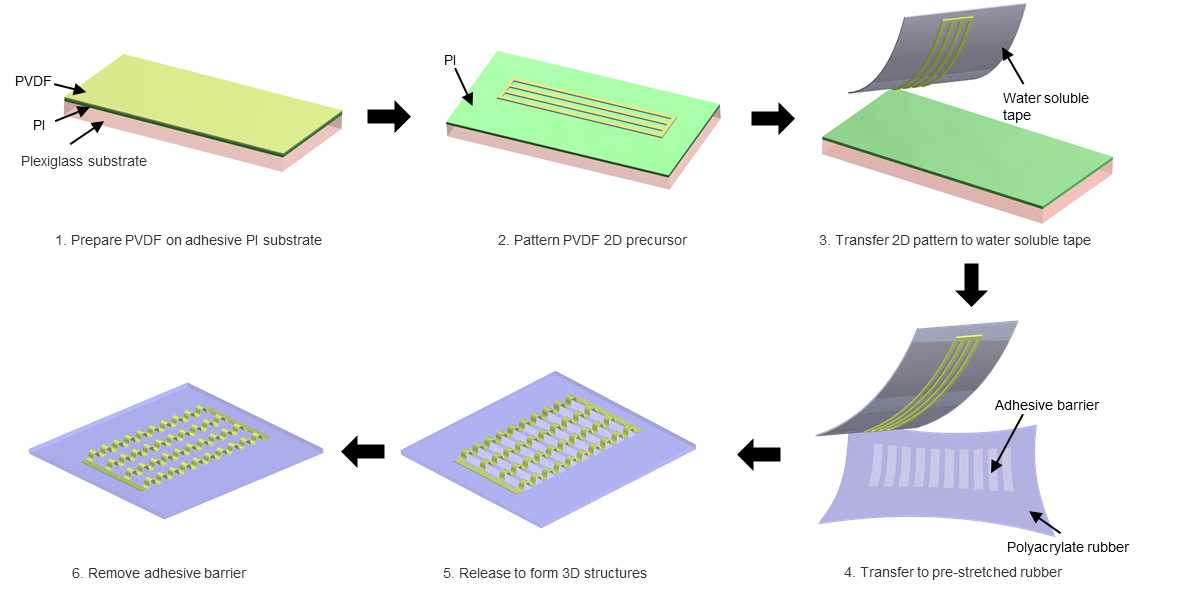


**Supplementary Figure 2. Fabrication scheme for 3D piezoelectric mesostructure.**

**
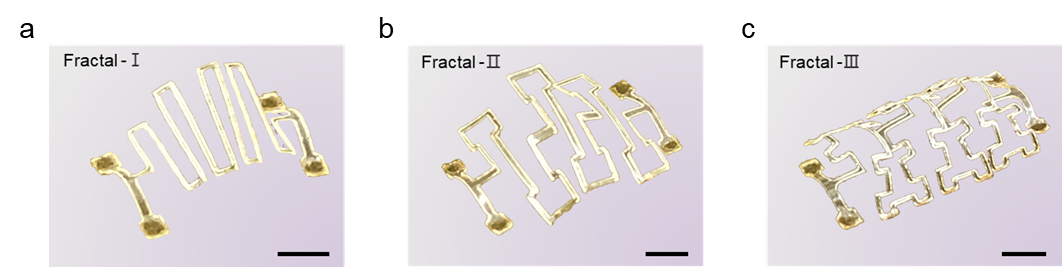
**

**Supplementary Figure 3. 3D fractal designs. a-c.** Optical imagine of various fractal manufactures, including first- (**a**) and second-order (**b**) fractal curves and a Hilbert geometry (c)**.** Scale bar, 2mm.


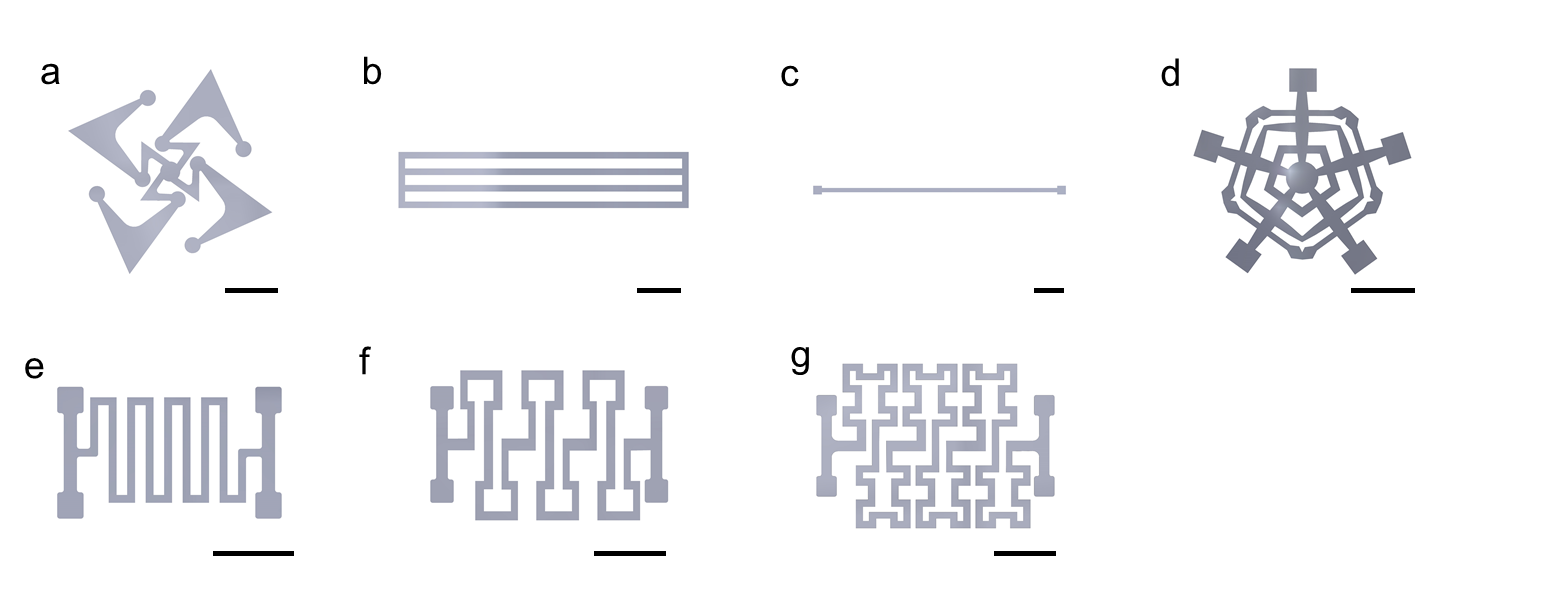


**S****upplementary Figure 4. 3D Precursor of the structures shown in Fig.1. a.** Flower**. b.** Wave. **c.** Spring. **d.** Flake**. e-g.** 3D Precursor of the fractal structures, first order (**e**), second orders (**f**) and Hilbert geometry (**g**). Scale bars, 3mm.

**
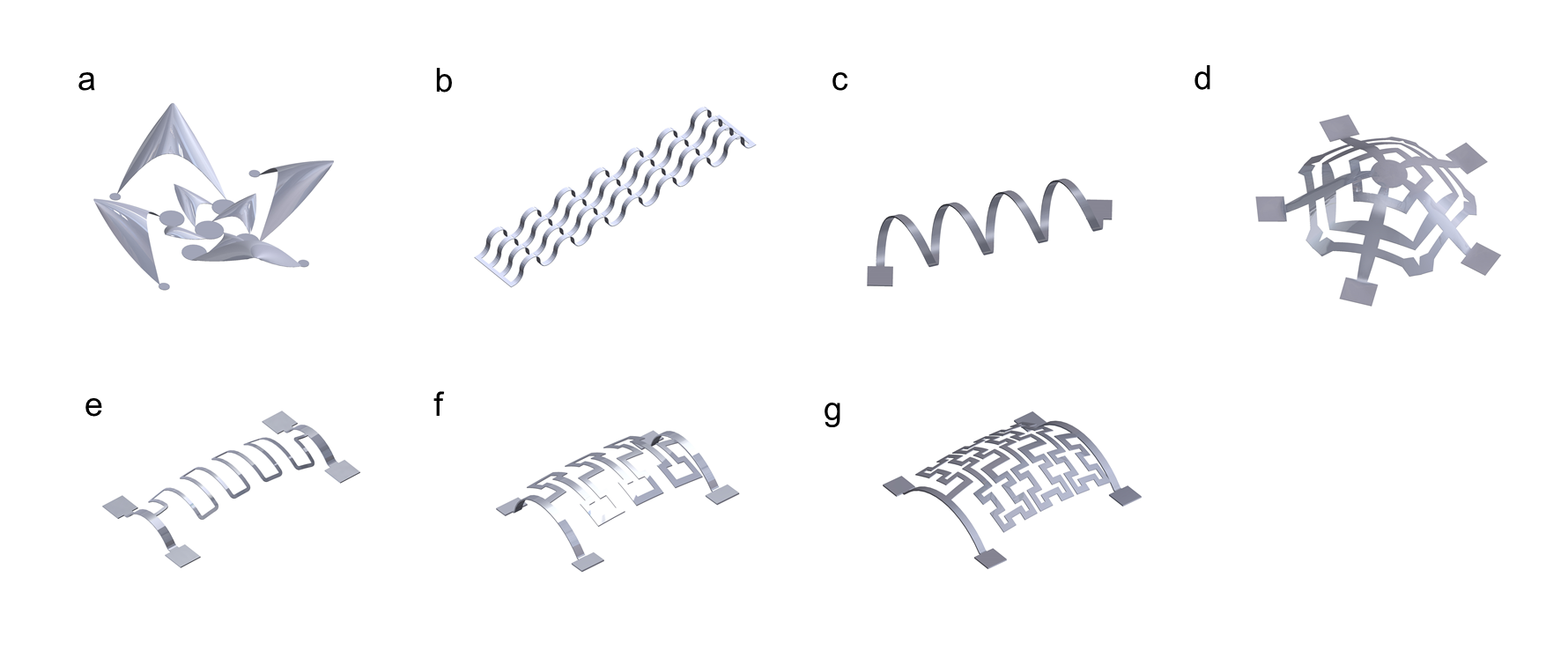
**

**Supplementary Figure 5.** **FEA predictions of 3D structures. a-c**. FEA prediction of the buckled shape for the structures in Fig. 1b, including flower (**a**), wave (**b**), spring (**c**), flake (**d**). **g-i.** FEA prediction of the buckled shape for the fractal structures, first order (**e**), second orders (**f**) and Hilbert geometry (**g**).


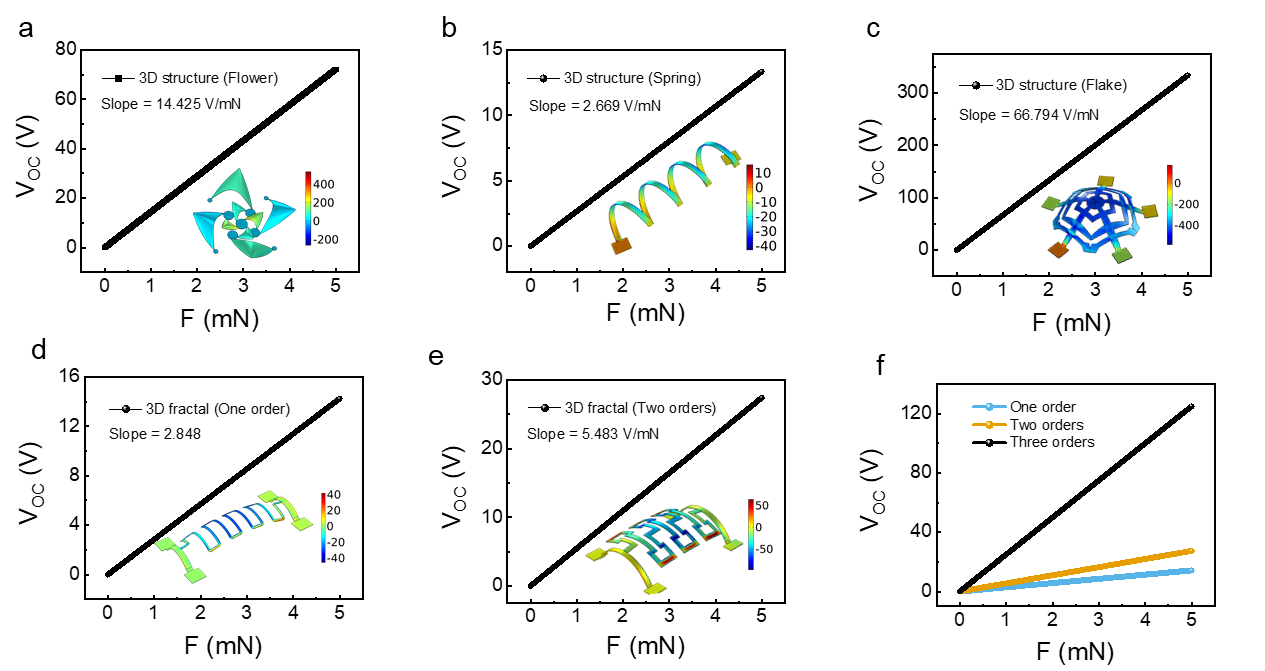


**Supplementary Figure 6. FEA results. a-c.** FEA of the 3D structures in Fig 1b**,** including flower (**a**), spring(**b**), flake (**c**). **d, e.** FEA of the fractal structures with first order (**d**) and Hilbert geometry (**e**)**.** **f.** Comparison of three types of fractal structure.

**
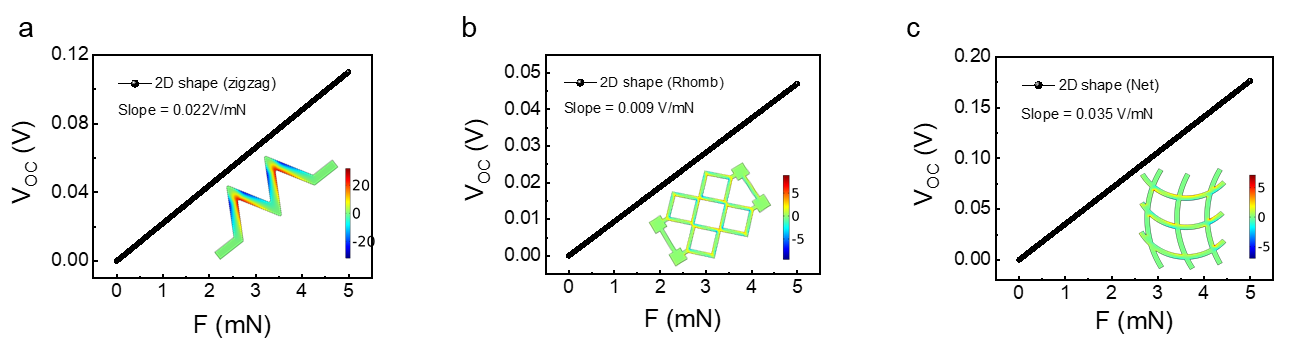
**

**Supplementary Figure 7. FEA of the 2D structures in Fig 1c. a.** Zigzag. **b.** Rhomb. **c.** Net**.**

**
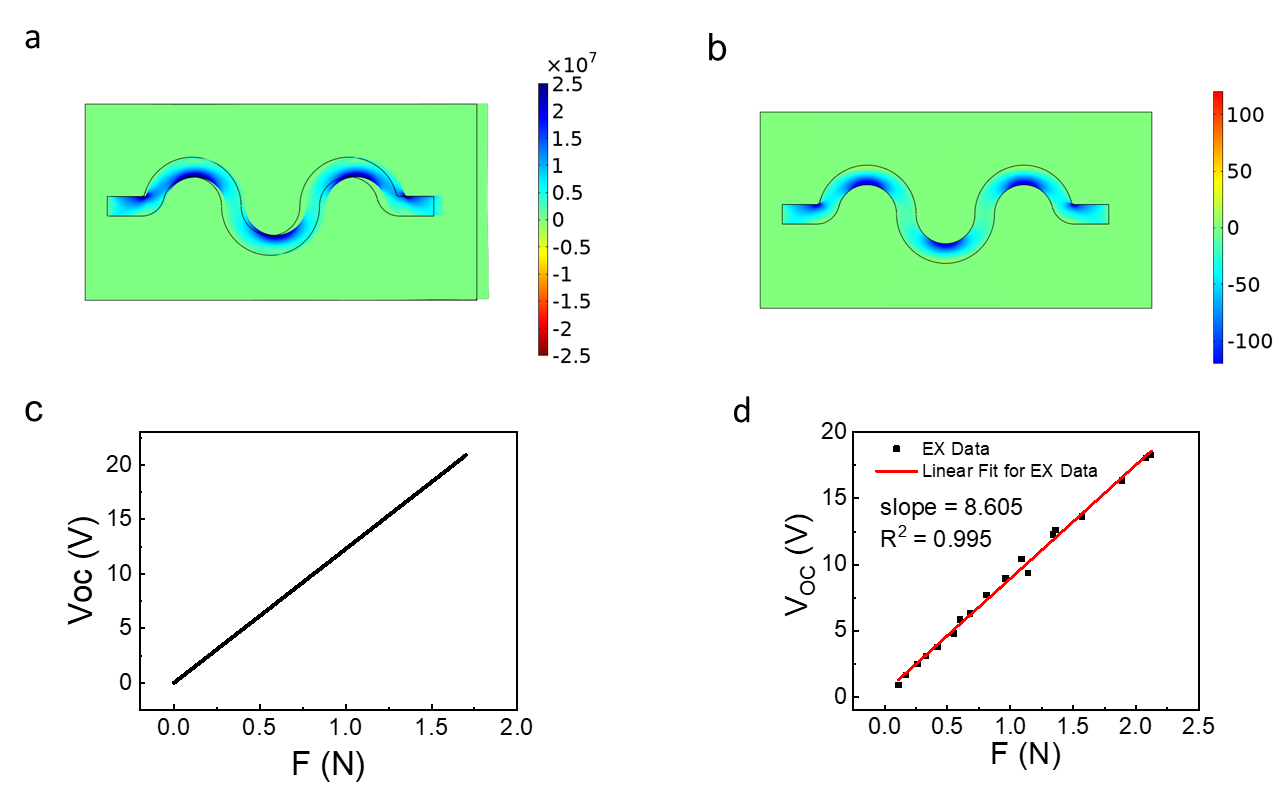
**

**Supplementary Figure 8. FEA of the serpentine structure with silicone rubber substrate. a.** Strain distribution. **b.** Surface charge distribution. **c.** FEA results**. d.** experiments results**.** The linear strain range of the typical devive is approximately 40%.

**
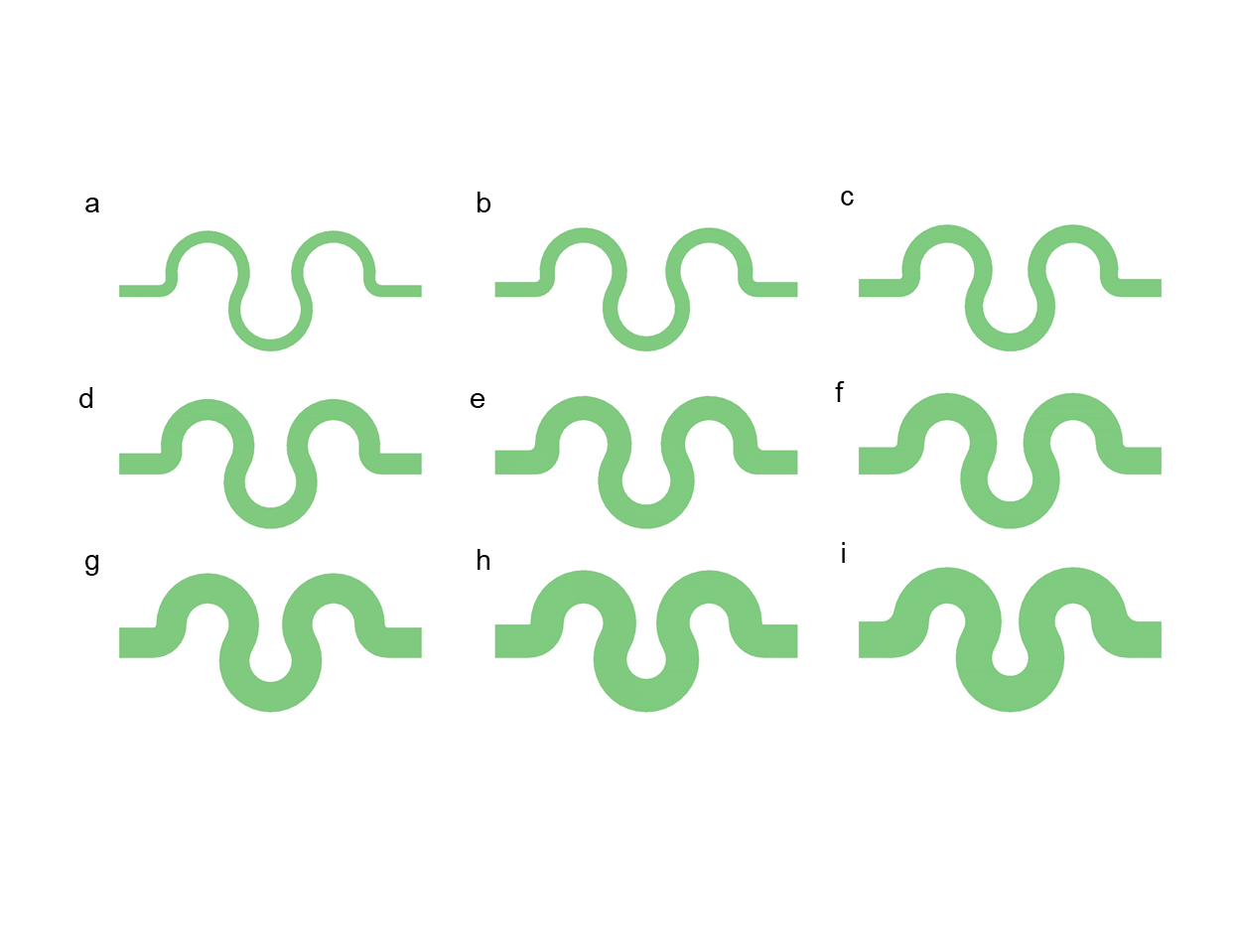
**

**Supplementary Figure 9. Illustration of different width structural design. Length, 5cm.**

**
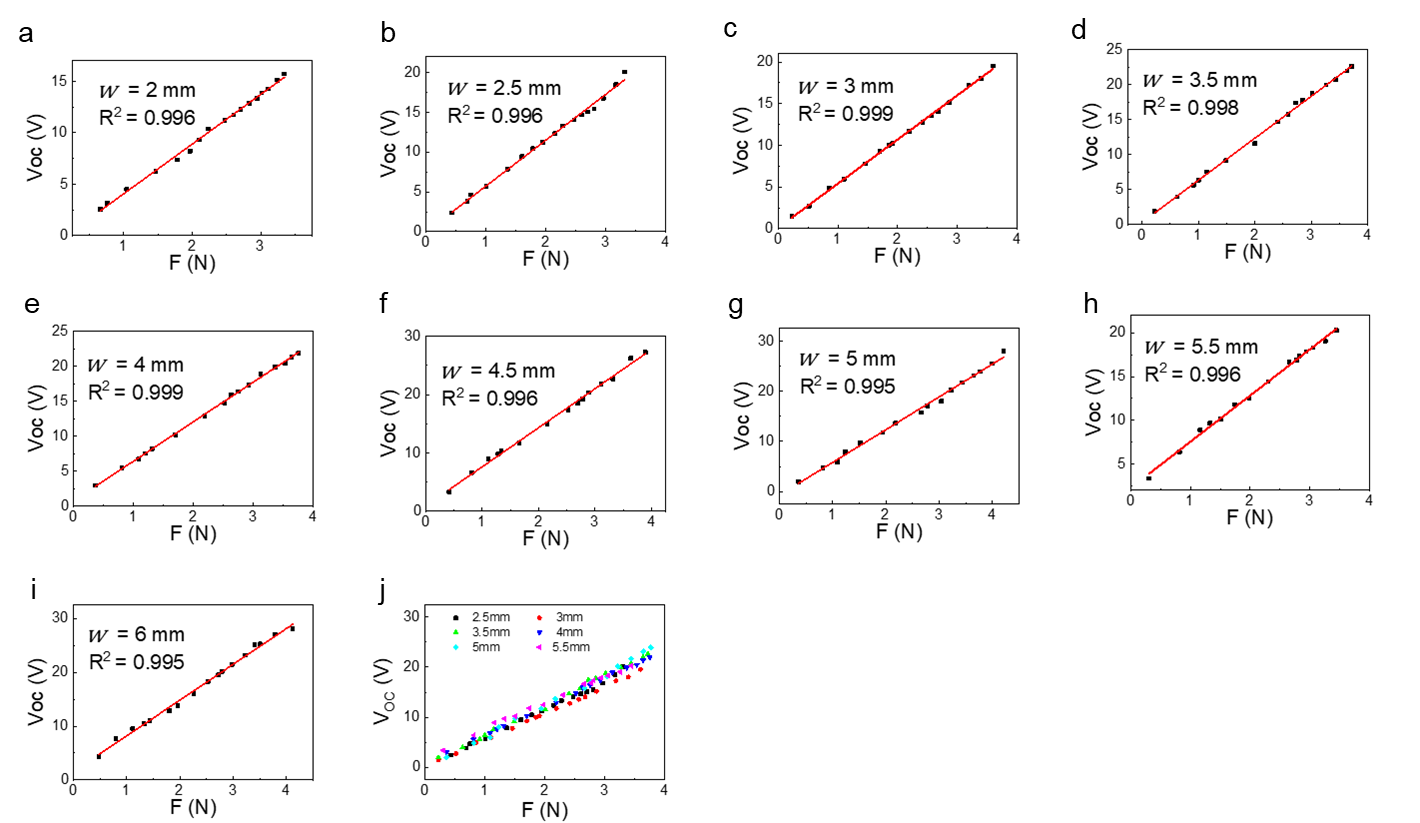
**

**Supplementary Figure 10. Experimental linear output graph with different width structure.**

**
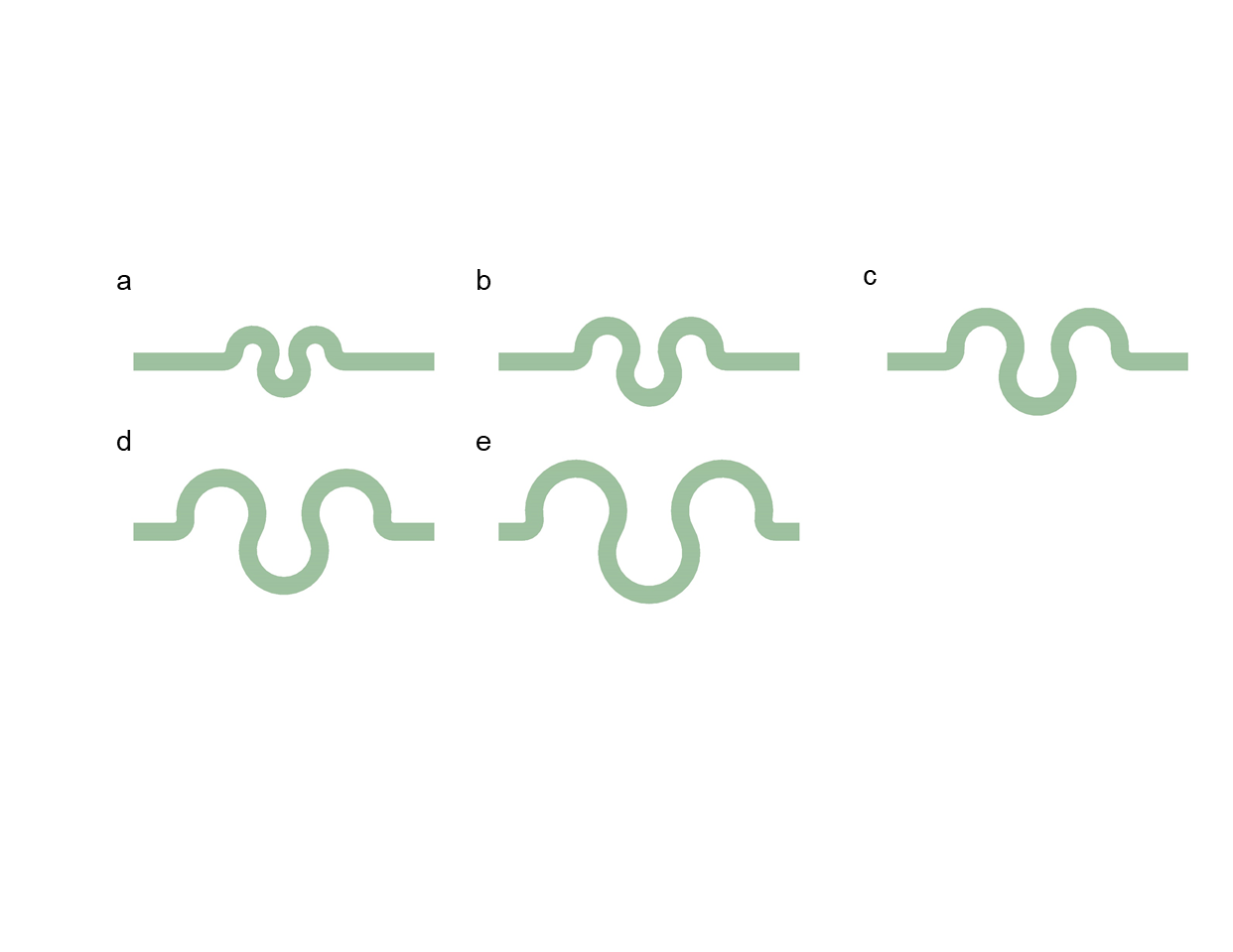
**

**Supplementary Figure 11. Illustration of different radius structural design in Fig. 2g. Length, 5cm.**

**
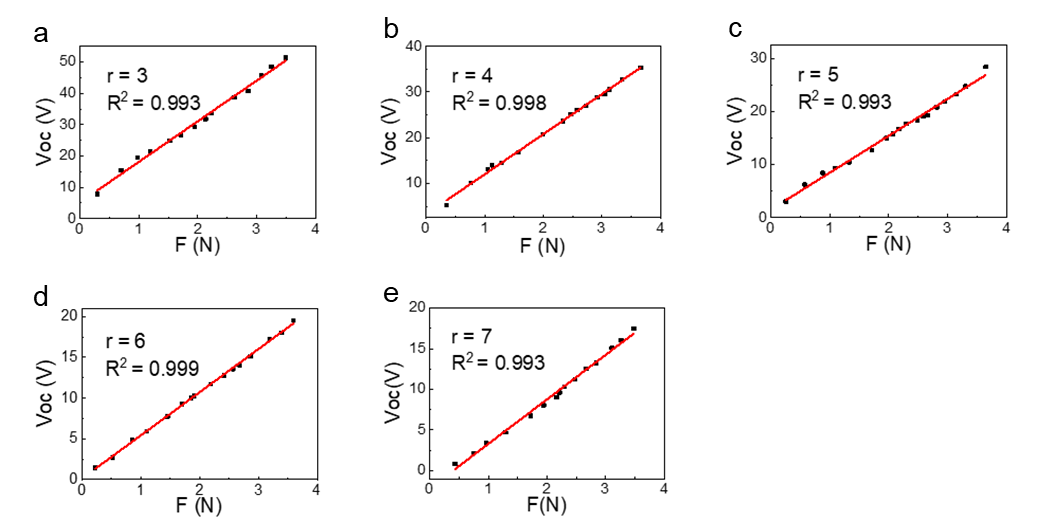
**

**Supplementary Figure 12. Experimental linear output graph with different radius in Fig. 2g**

**
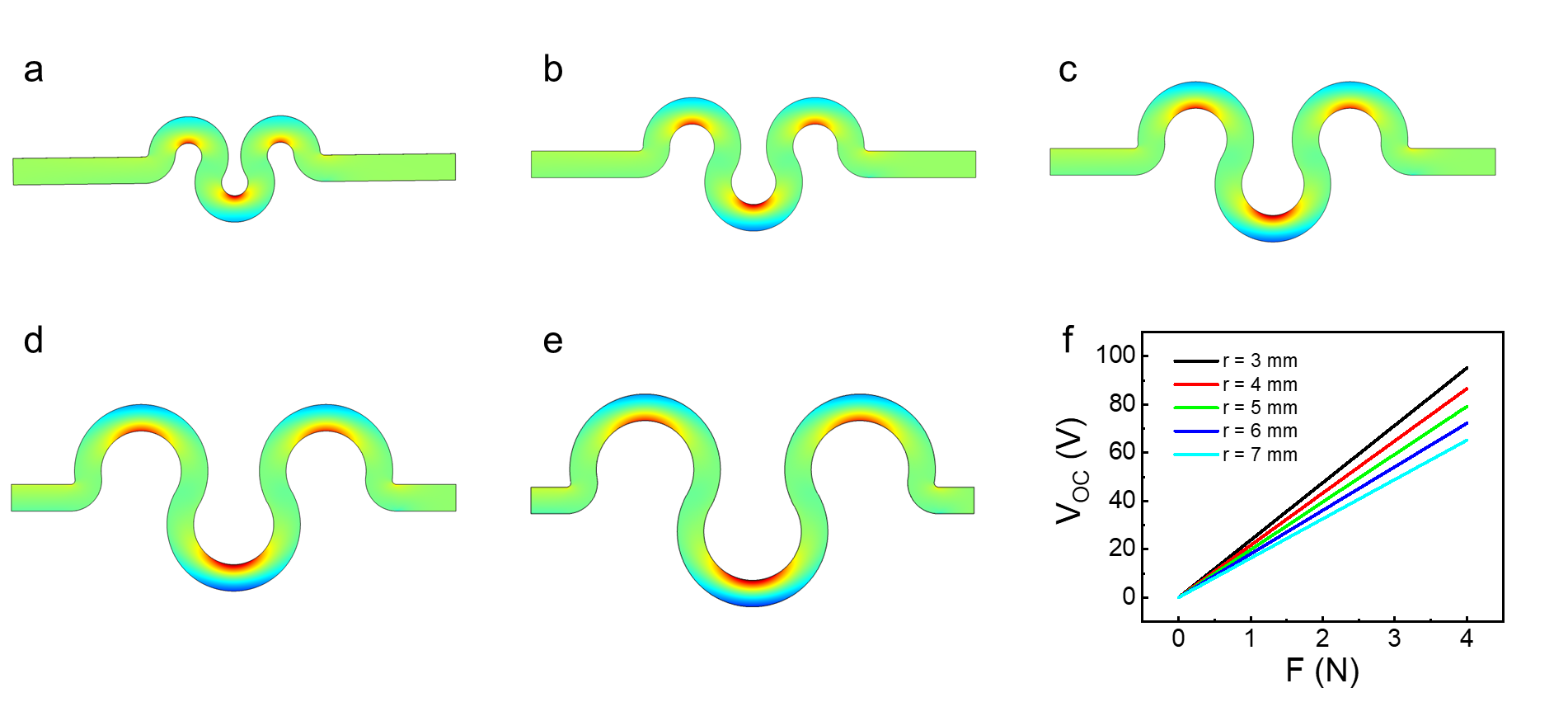
**

**Supplementary Figure 13. FEA results of different angles in Fig. 2g. a-e.** Surface potential distribution of various radius, including 3 mm (a), 4mm (b), 5mm (c), 6mm (d), 7mm (e). **f.** Comparation of surface potential changes with load at different radius. Length, 5cm.

**
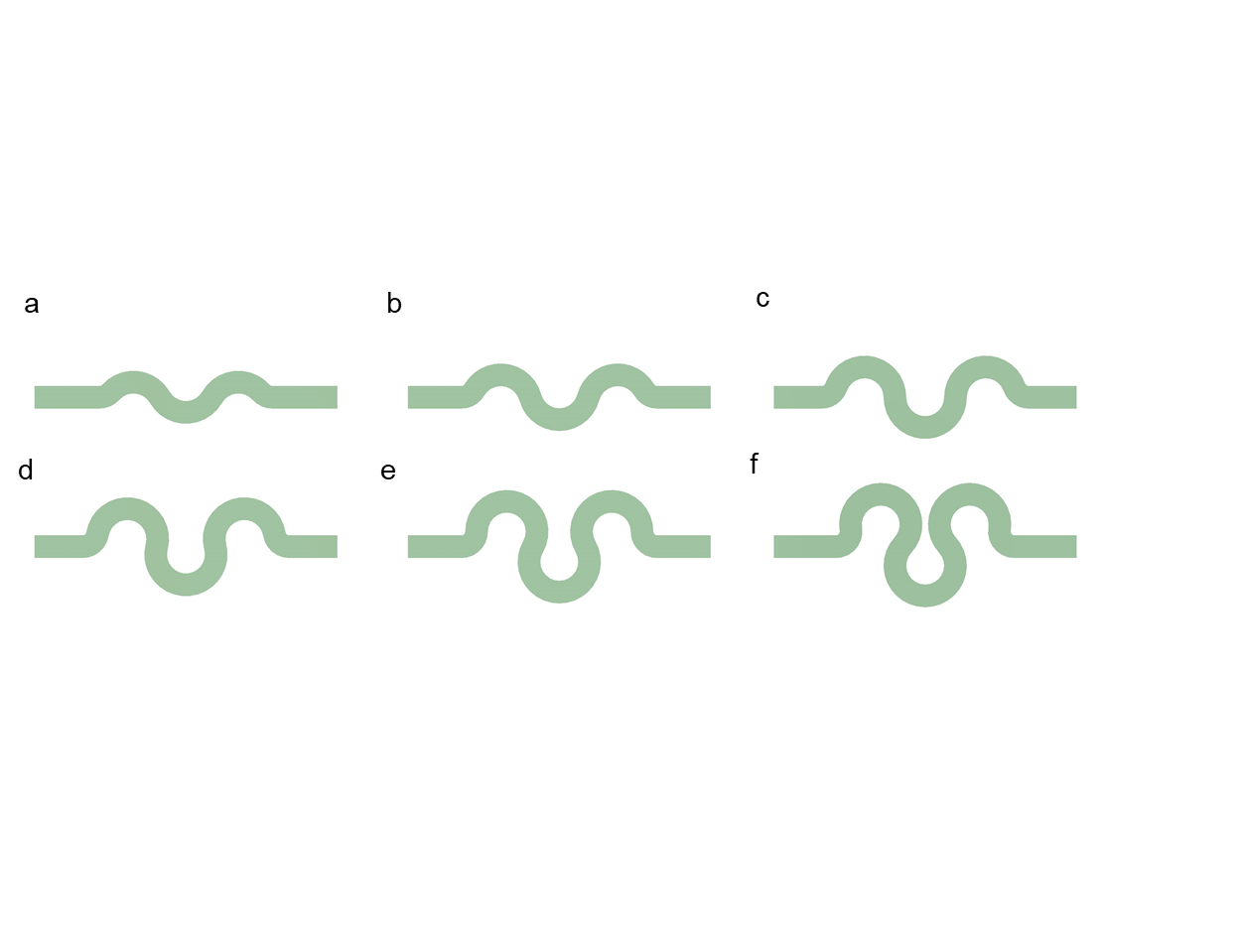
**

**Supplementary Figure 14. Illustration of different angle structural design in Fig. 2h. Length, 5cm.**

**
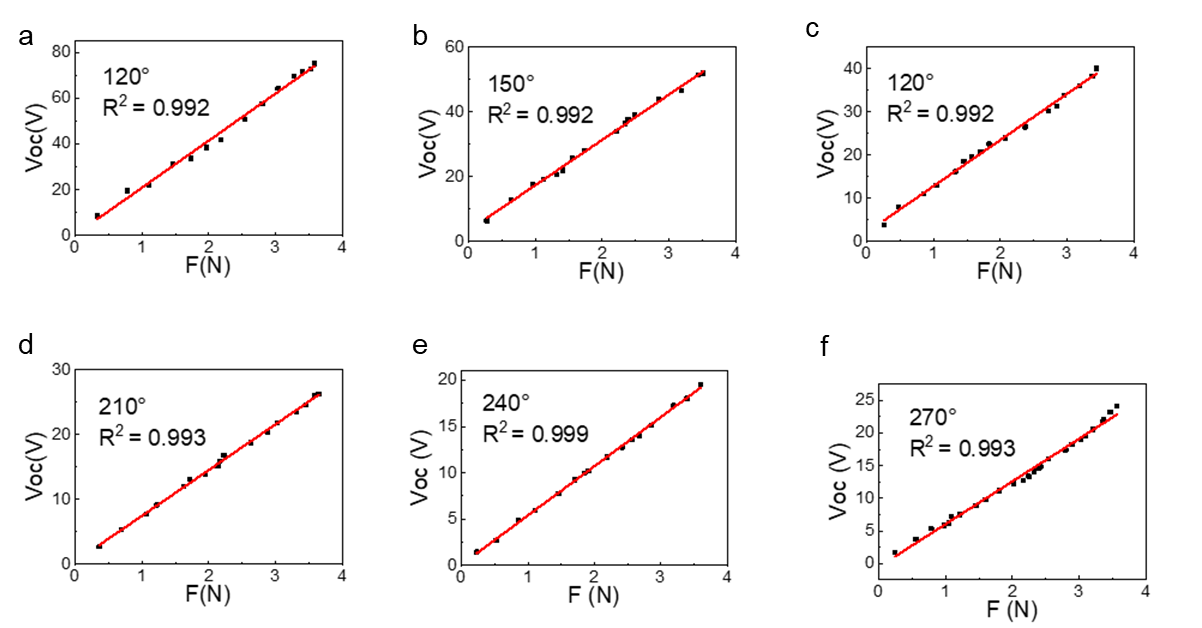
**

**Supplementary Figure 15. Experimental linear output graph with different angles in Fig. 2h.**


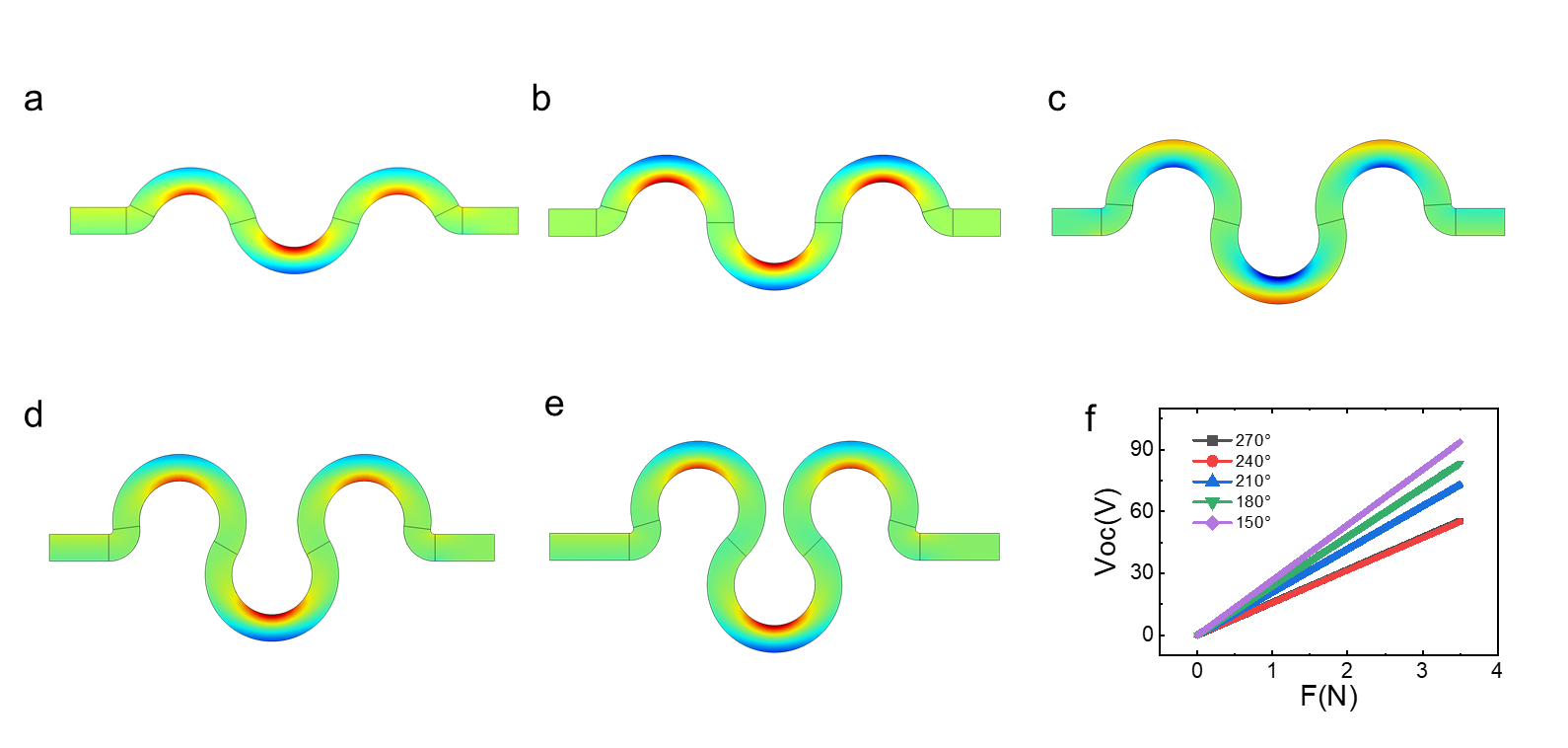
**Supplementary Figure 16. FEA results of different angles in Fig. 2h. a-e.** Surface potential distribution of various angles, including 150° (a), 180° (b), 210° (c), 240° (d)270° (e). **f.** Comparation of surface potential changes with load at different angles. Length, 5cm.


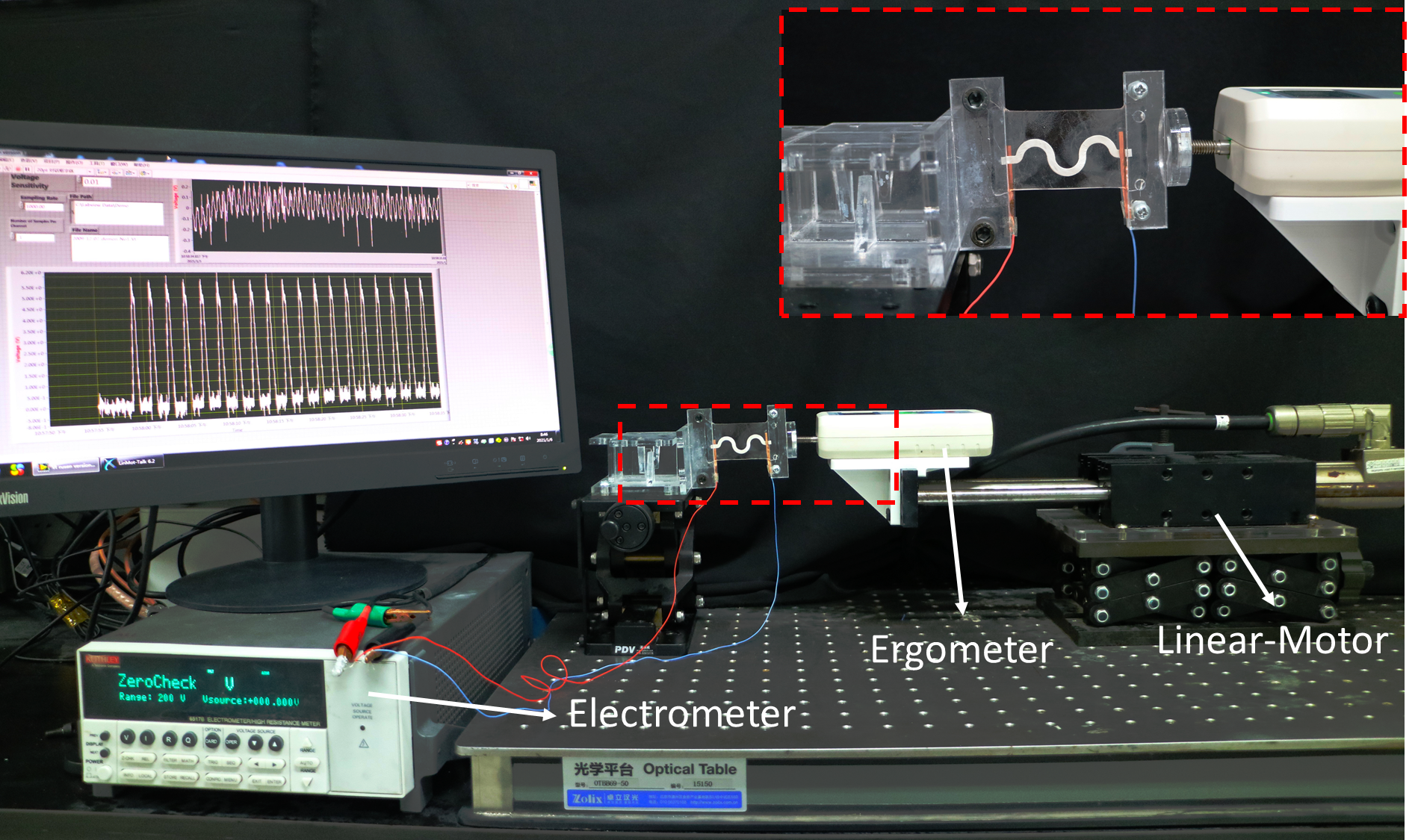


**Supplementary Figure 17. Quantitative mechanized measurement method of the patch.**

**
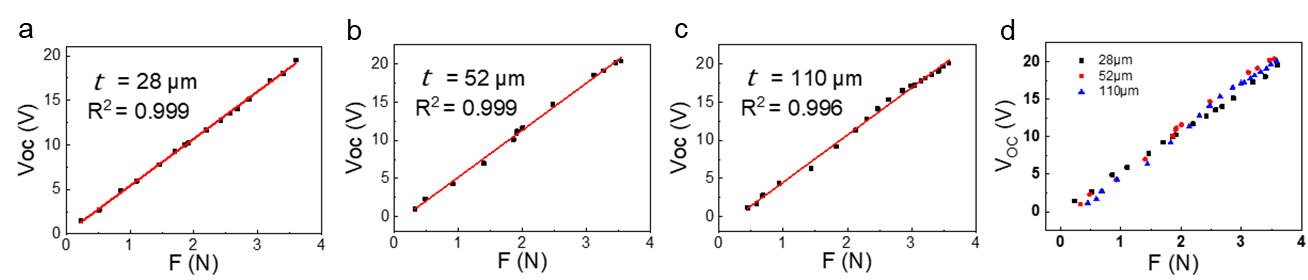
**

**Supplementary Figure 18.**  **Variation of open circuit voltage with experimental load under different thickness. a-c.** Linear output graph with different thickness structure, including 28μm (a), 52μm (b) and 110μm (c). **d.** Comparation of open-circuit voltage changes with load at different thickness.

**
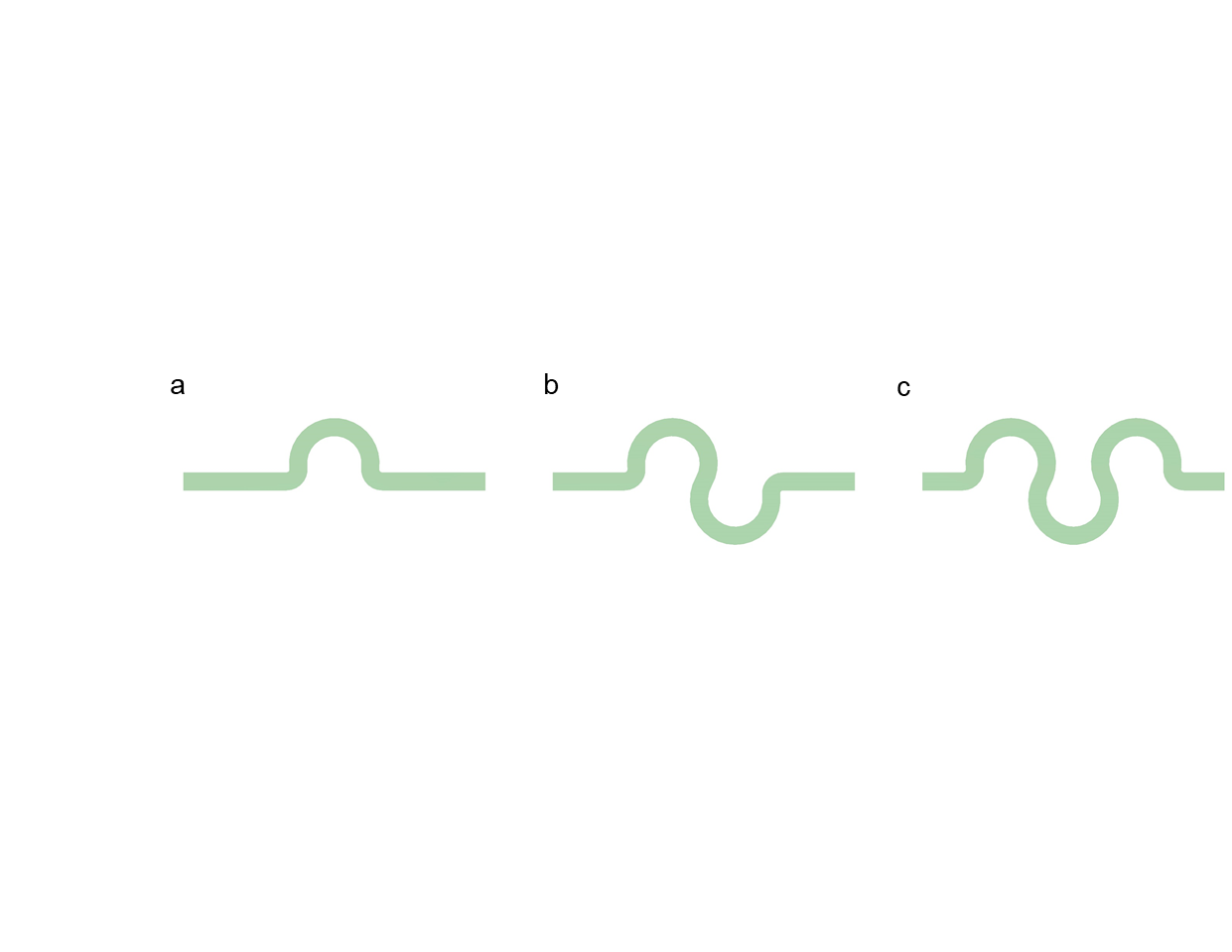
**

**Supplementary Figure 19. Illustration of different numbers of the arc structural design in Fig. 2i. Length, 5cm.**

**
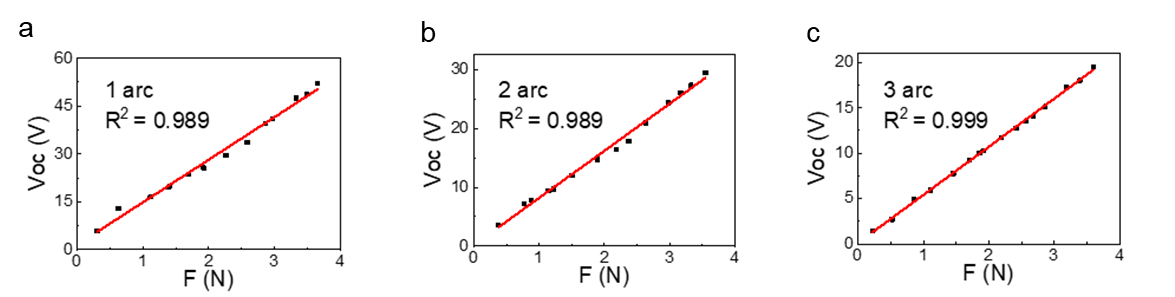
**

**Supplementary Figure 20. Experimental linear output graph with different numbers of the arc structure in Fig. 2i.**

**
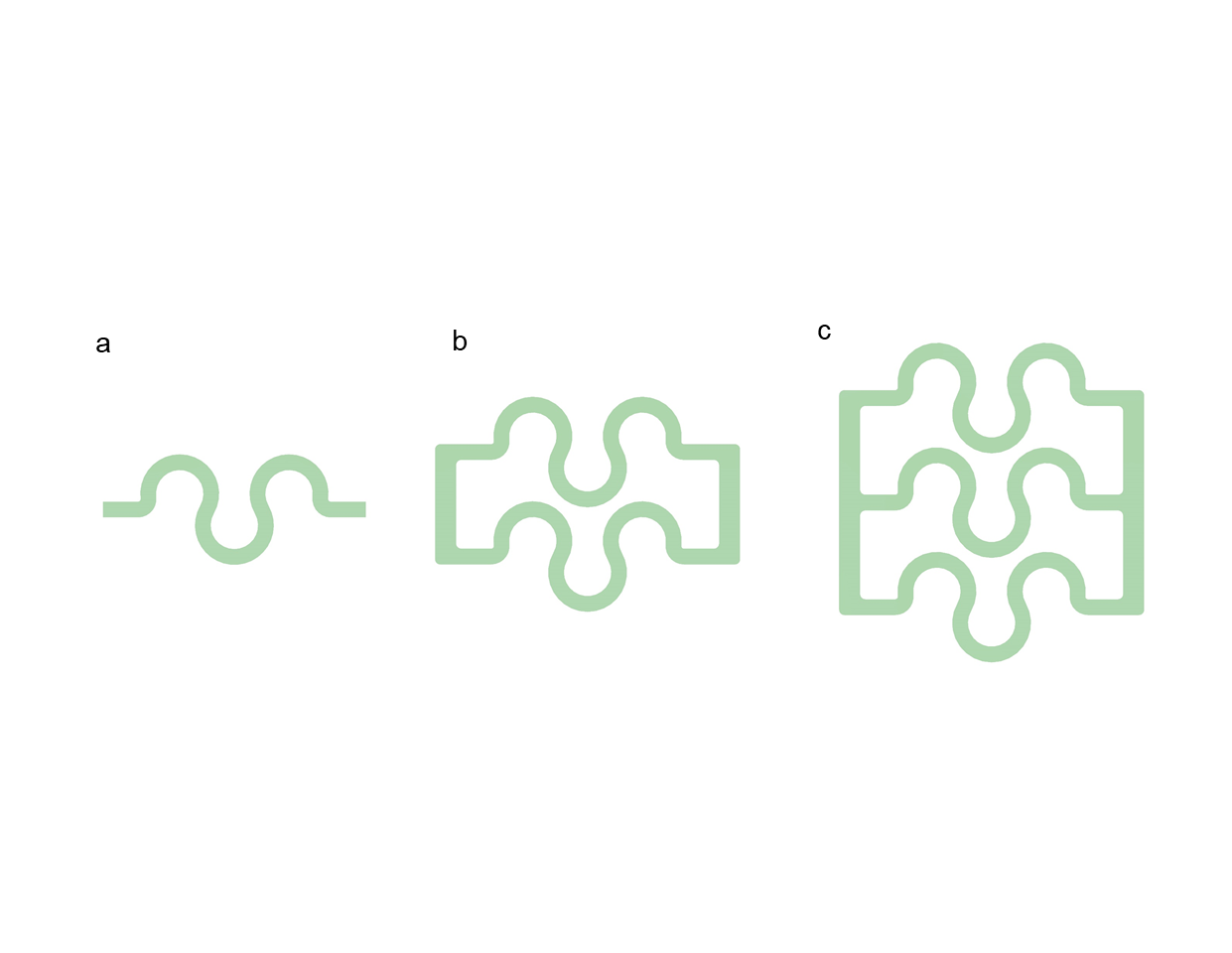
**

**Supplementary Figure 21. Illustration of different numbers of the units’ structural design in Fig. 2j. Length, 5cm.**

**
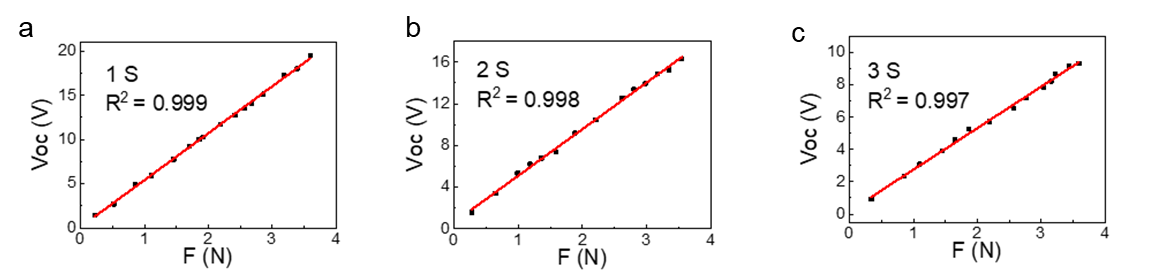
**

**Supplementary Figure 22. Experimental linear output graph with different numbers of the unit in Fig. 2j.**


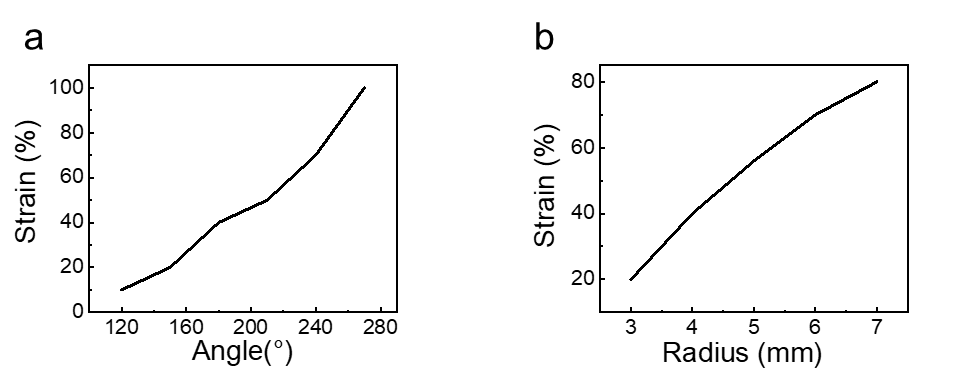


**Supplementary Figure 23.** **Relationship of Radius and angle vs. strain to illustrate the stretchability of the structures in Fig. 2.**

**Supplementary Figure 24. Comparison of open circuit voltages at various speeds.**

**
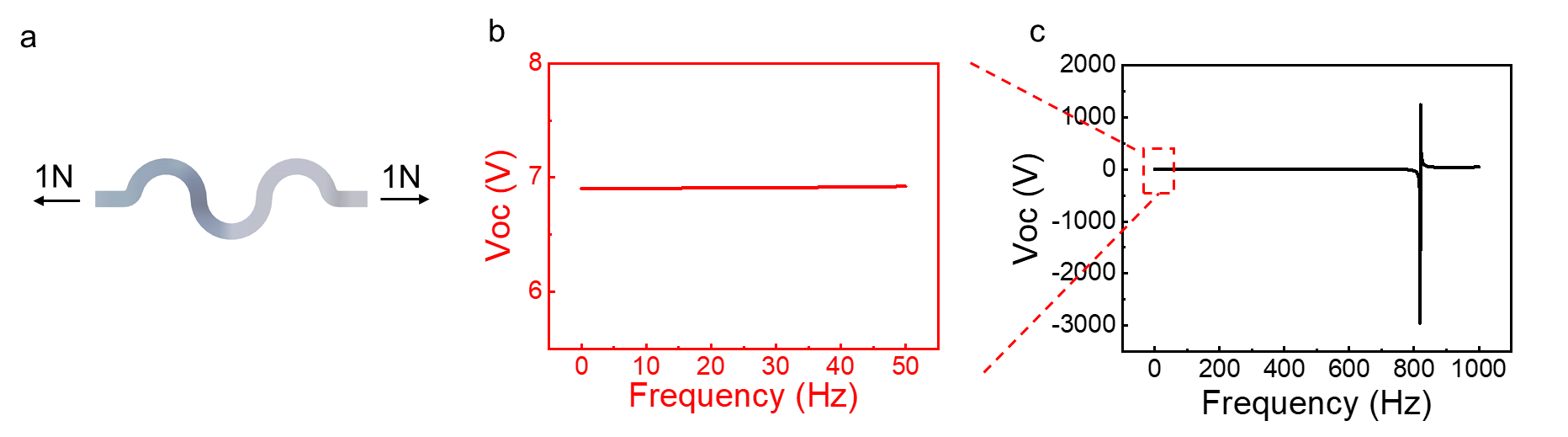
**

**Supplementary Figure 25.** **Simulation diagram of frequency response characteristics under 1N force. a.** Schematic diagram of force action. **b.** Low frequency output under 1N force. **c.** Voltage vs. frequency graph.

**
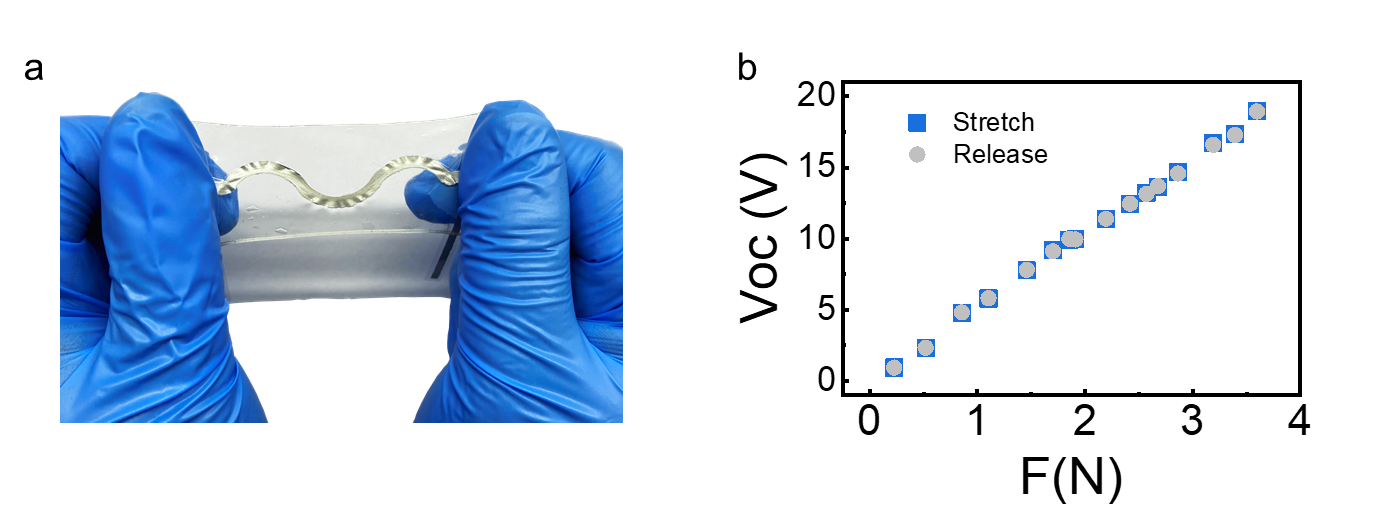
**

**Supplementary Figure 26. Comparison chart of stretch and resilience performance. a.** Schematic of stretching. **b.** Comparison of potential difference between stretch and rebound under various forces.

**Supplementary Figure 27.** **Cyclic measurement of the device under 20% of the tensile.**


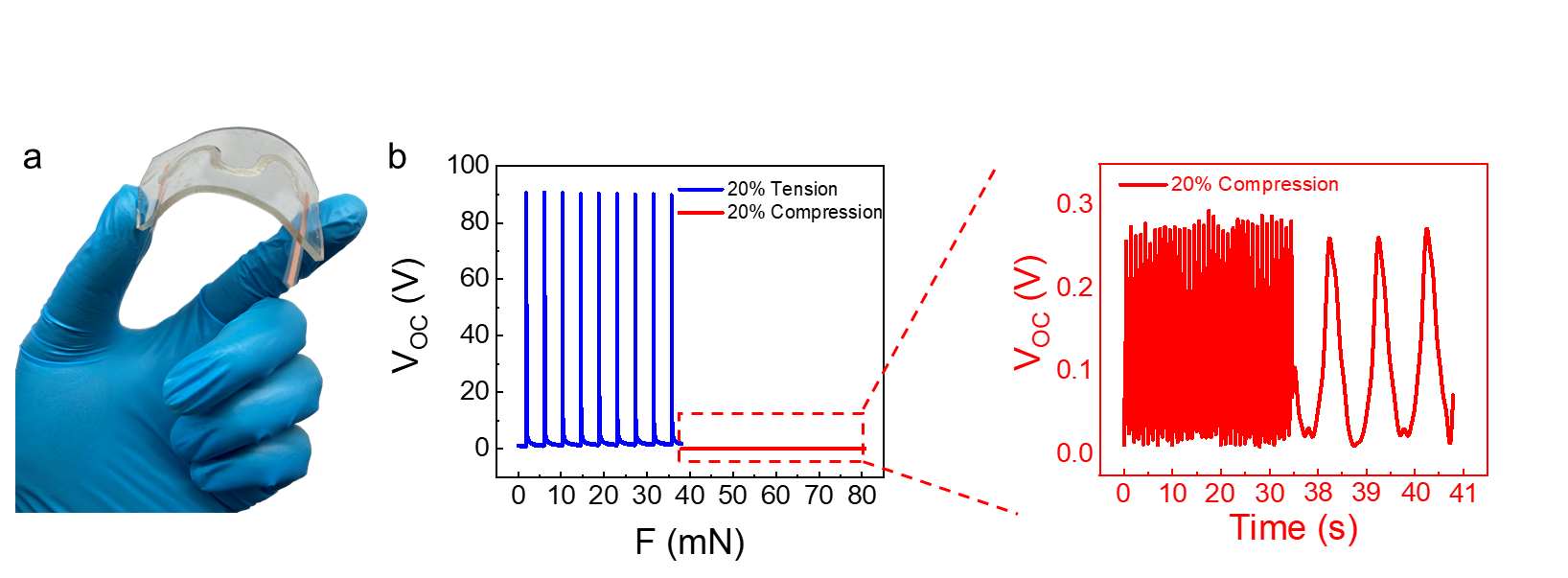


**Supplementary Figure 28.** **Comparison of open circuit voltage between stretch and bending. a.** Schematic of bending. **b.** Output comparison under 20% tension and compression, the red enlarged imagine shows the open-circuit voltage of the patch at 20% compression.

**
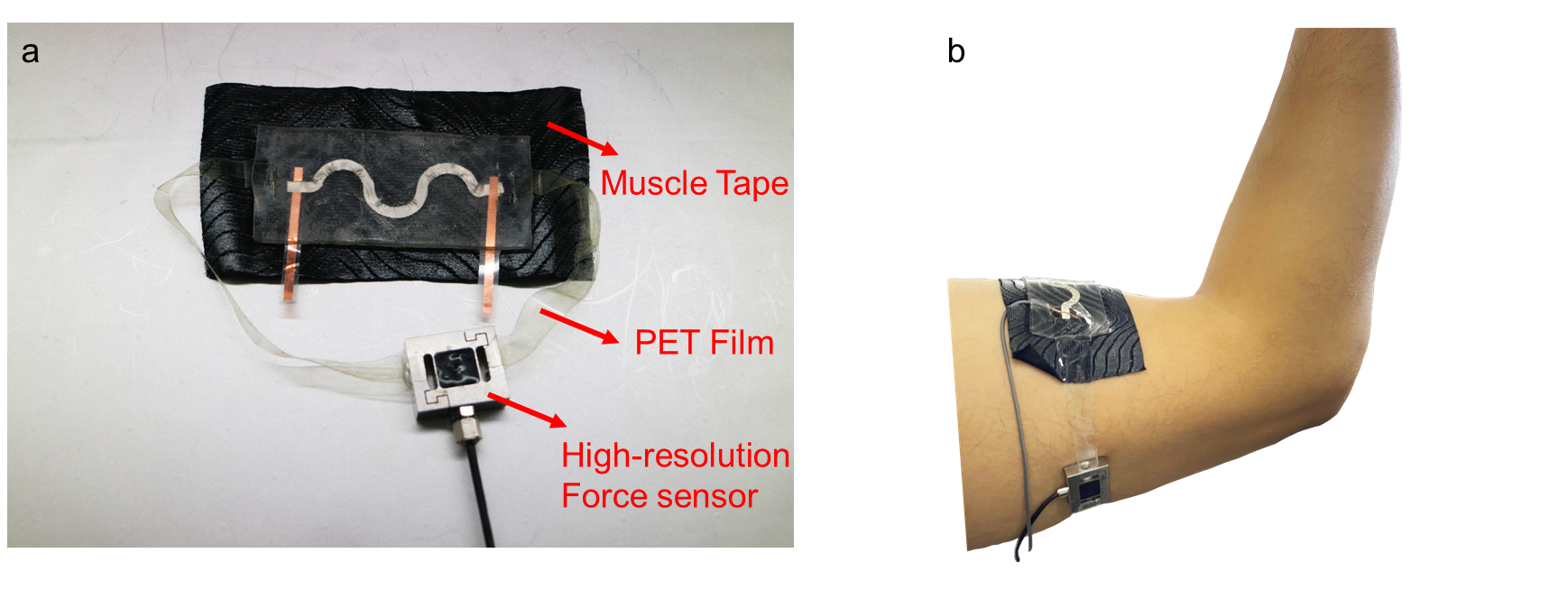
**

**Supplementary Figure 29.** **Diagram of the patch and commercial sensors in series. a.** Illustration of the connection between thepatch and commercial sensors. **b.** The patch and commercial sensors are fixed on the biceps for tandem measurement.

**
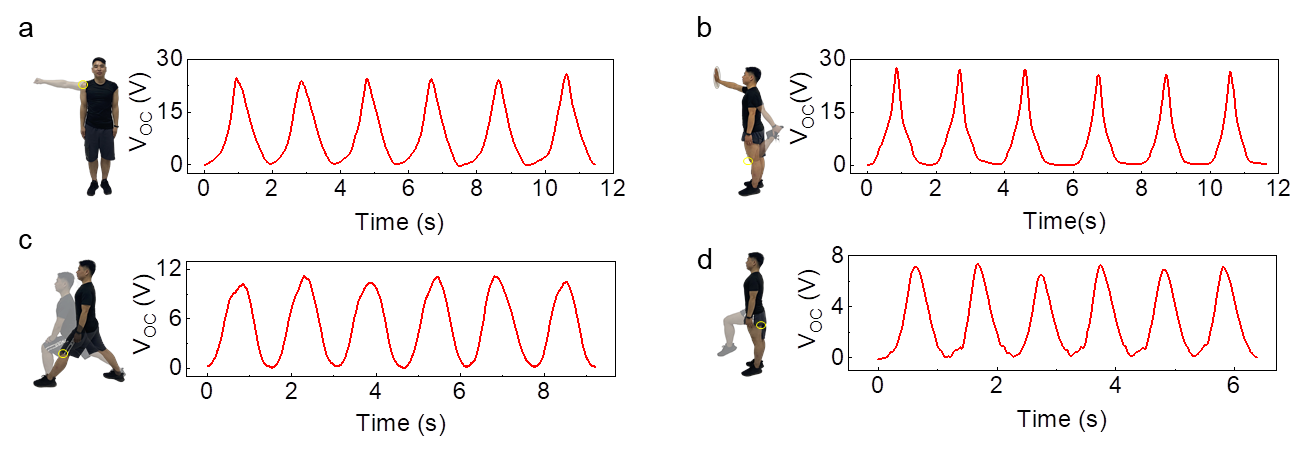
Supplementary Figure 30.** Open-circuit voltage of the patch sticking to the deltoid (**a**), quadriceps femoris (**b**), anterior knee ligament (**c**) and hamstrings (**d**).

**
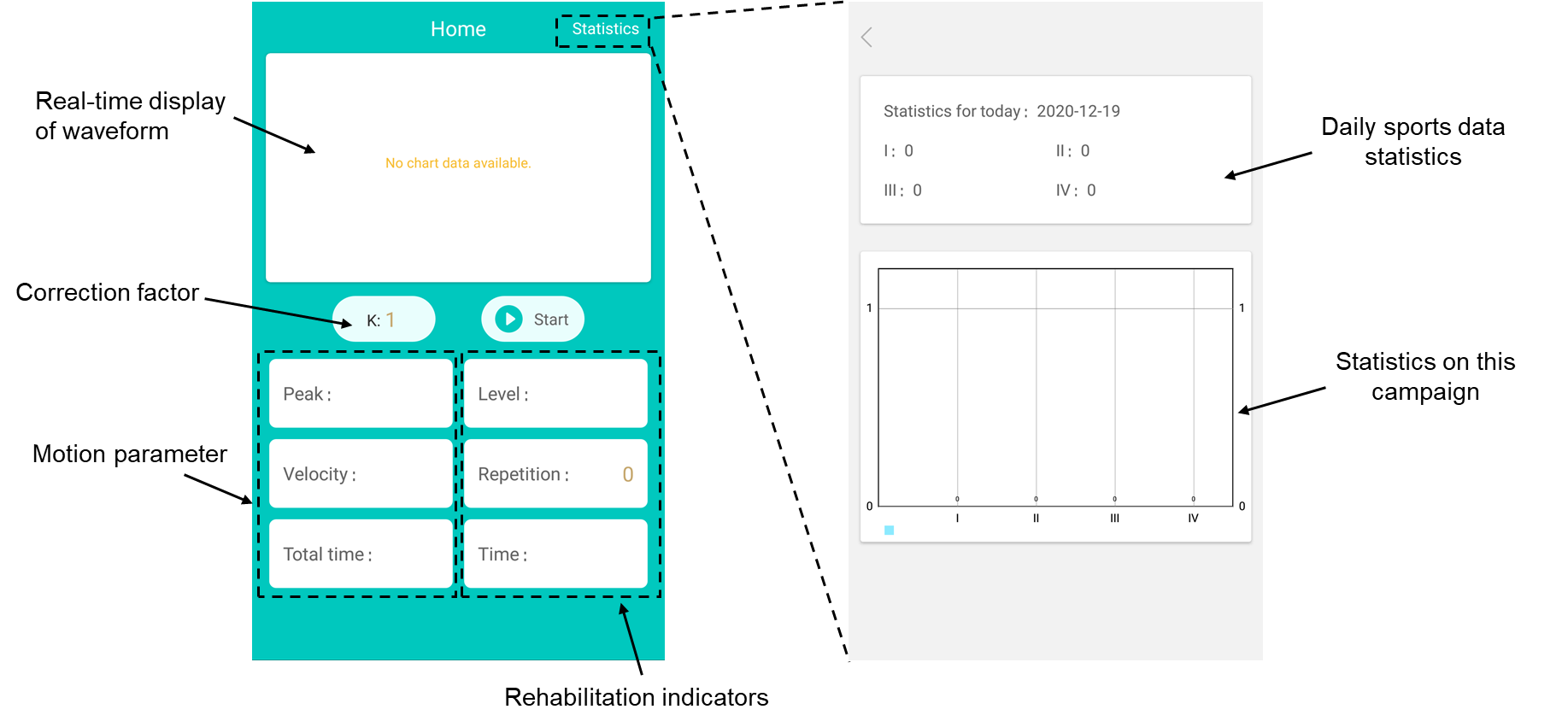
**

**Supplementary Figure 31. APP interface that can provide feedback information.**

**
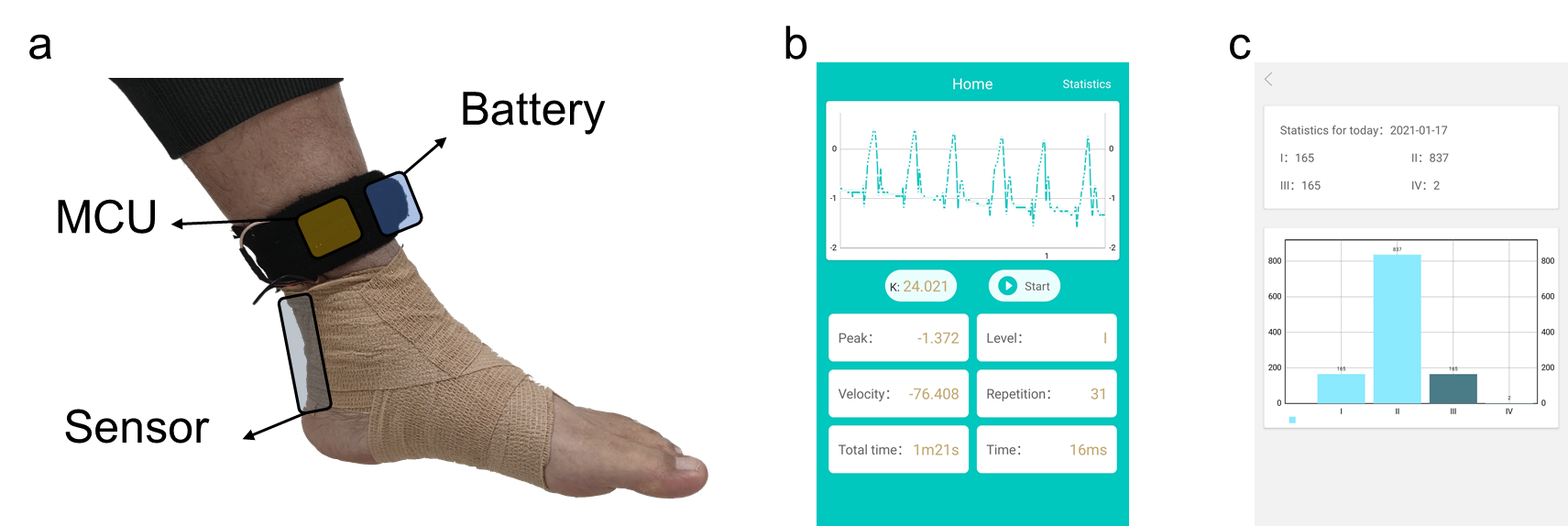
**

**Supplementary Figure 32. Simulation of paretic gait. a.** Diagram of gait simulation method**. b, c.** APP screen shot of paretic gait monitoring.
